# Single-cell-type quantitative proteomic and ionomic analysis of epidermal bladder cells from the halophyte model plant *Mesembryanthemum crystallinum* to identify salt-responsive proteins

**DOI:** 10.1186/s12870-016-0797-1

**Published:** 2016-05-10

**Authors:** Bronwyn J. Barkla, Rosario Vera-Estrella, Carolyn Raymond

**Affiliations:** Southern Cross Plant Science, Southern Cross University, Lismore, NSW 2480 Australia; Instituto de Biotecnología, Universidad Nacional Autónoma de México, Cuernavaca, MOR México

**Keywords:** Proteomics, Trichome, Salinity, Salt tolerance, Crassulacean acid metabolism (CAM), Ionomics, Chloride, Sodium, V-ATPase, Single cell-type

## Abstract

**Background:**

Epidermal bladder cells (EBC) are large single-celled, specialized, and modified trichomes found on the aerial parts of the halophyte *Mesembryanthemum crystallinum*. Recent development of a simple but high throughput technique to extract the contents from these cells has provided an opportunity to conduct detailed single-cell-type analyses of their molecular characteristics at high resolution to gain insight into the role of these cells in the salt tolerance of the plant.

**Results:**

In this study, we carry out large-scale complementary quantitative proteomic studies using both a label (DIGE) and label-free (GeLC-MS) approach to identify salt-responsive proteins in the EBC extract. Additionally we perform an ionomics analysis (ICP-MS) to follow changes in the amounts of 27 different elements. Using these methods, we were able to identify 54 proteins and nine elements that showed statistically significant changes in the EBC from salt-treated plants. GO enrichment analysis identified a large number of transport proteins but also proteins involved in photosynthesis, primary metabolism and Crassulacean acid metabolism (CAM). Validation of results by western blot, confocal microscopy and enzyme analysis helped to strengthen findings and further our understanding into the role of these specialized cells. As expected EBC accumulated large quantities of sodium, however, the most abundant element was chloride suggesting the sequestration of this ion into the EBC vacuole is just as important for salt tolerance.

**Conclusions:**

This single-cell type omics approach shows that epidermal bladder cells of *M. crystallinum* are metabolically active modified trichomes, with primary metabolism supporting cell growth, ion accumulation, compatible solute synthesis and CAM. Data are available via ProteomeXchange with identifier PXD004045.

**Electronic supplementary material:**

The online version of this article (doi:10.1186/s12870-016-0797-1) contains supplementary material, which is available to authorized users.

## Background

Single-cell-type analysis is a powerful experimental approach, allowing for the capture of information from specific cell types that would normally be lost due to the heterogeneity of cells in a tissue, giving us greater insight into the role of specialized cells. In plants, successful single cell type analysis has been undertaken for only a handful of cell types, including pollen grains, but also, due to ease of isolation, cells of the epidermis, such as root hairs, guard cells and trichomes [1]. Trichomes are highly differentiated cell types found on the aerial epidermis of most plants. These specialized cells vary morphologically and functionally, with roles in plant defence, stress tolerance, water collection, seed dispersal and leaf structure. They can range from simple unicellular hair-like extensions to multicellular complex appendages [2, 3]. They are classified as non-secreting or glandular-secreting trichomes; the latter can secrete a vast array of substances including lipophilic compounds, proteins, ions, sugars and secondary plant products [4, 5]. Halophyte plant species have evolved several different types of trichomes ranging from bi- or multi-cellular glands of the Poaceae, which actively excrete salt [6], to non-glandular (non-secreting) trichomes called epidermal bladder cells (EBC). These EBC are attached to either the epidermis via stalk cells, as in the Chenopodiaceae [7], or stalk-less as in the Mesembryanthemaceae [8]. In the halophyte *Mesembryanthemum crystallinum* these single celled EBC are present on leaves, stems and flower buds. Cell morphology changes with plant age and metabolic/stress state of the plant. In young plants the EBC are small and flattened to the leaf surface and stem, whereas in adult plants that are undergoing Crassulacean acid metabolism (CAM), and particularly those exposed to salt, the cells swell up and appear as liquid filled balloons. On average, the diameter of EBC can be 1 mm with an average cell volume of 500 nl; although volumes in excess of 5 μl have been reported [8, 9].

Early work on the physiology of EBC in *M. crystallinum* concluded that these cells were predominantly involved in water storage during times of reduced water availability [10]. However, we now know that they are also substantial stores for sodium ions. EBC have been shown to accumulate as much as 1.2 M Na^+^ which is thought to be sequestered into the large central vacuole [8, 11]. Evidence that EBC are essential for salt tolerance of *M. crystallinum*, comes from studying a mutant that had reduced numbers of these specialized cells [12]. Mutant plants showed diminished Na^+^ accumulation capacity, reduced leaf and stem water content and a significant reduction in seed number, however the gene involved was not identified.

Initial proteomic profiling of the EBC extract isolated from salt-treated adult *M. crystallinum* plants by single cell sampling techniques and shot-gun LC-MS/MS was only able to identify 84 proteins at high confidence. These belonged to diverse functional classes, including proteins involved in ion and water homeostasis, but also photosynthesis related proteins and proteins associated with CAM [9]. Understanding which proteins are critical and central for bladder cell function and adaptation to salt stress can only be obtained by direct comparisons between EBC from untreated plants and plants that have been salt-treated. In this study, we carry out large-scale complementary quantitative proteomic studies using both a label and label-free approach to identify salt-responsive proteins in the EBC extract. Using these approaches, we were able to identify 438 proteins at high confidence and show significant changes between treatment conditions in 54 of these. In order to confirm these results a number of the proteins were then validated by western blot analysis. In parallel, an ionomics analysis was carried out to determine the ion profile of the bladder cells and how this may change under salinity stress with the accumulation of sodium in these cells. These results, combined with our previous transcriptomics and metabolomics profiling data, allow for an integrated view of the adaptive responses occurring in the bladder cells to salt-treatment.

## Results

### Quantitative proteomic analysis

To identify salt-responsive proteins in EBC extracts, a study combining complementary 2D-DIGE and 1D-PAGE with label-free LC-MS/MS (GeLC-MS/MS) based quantification methods was performed to compare proteins in extract from salt-treated plants to EBC extract from untreated control plants with the aim to maximize the coverage of the proteome. The use of distinctive approaches, which exploit alternative technologies requiring unique sample handling procedures, helped to obtain a greater coverage of the salt-responsive proteome.

Spot maps from 2D-DIGE gels resolved on average 1384 spots in each of the four gels following automatic detection in the DIA module of the Decyder 6.5 software; a representative image of one of the gels is shown in Additional file 1A. The majority of the protein spots were observed within a pH range of 4 to 7, but there were also numerous protein spots in the acidic and basic regions of the gels, including a large smear of unresolved protein which ran at the basic limit (pH 11) of the IEF gel. The BVA module of the Decyder 6.5 software was used for inter-gel matching and was performed by means of automatically land marking spots in the Cy2 internal standard images from each gel and then manually confirming matched spots. To ensure robust matching a minimum of 30 spots were landmarked and confirmed in each of the gels. Statistical analysis was then performed on matched spots to identify differentially abundant proteins between the control and salt-treated EBC samples. This is performed automatically by normalizing spot volumes against the internal standard. To be included for downstream LC-MS/MS analysis statistically significant, differentially abundant proteins were required to fulfil several criteria: (1) Spots must be present and matched in all spot maps from all gel images (12/12). (2) Spots which showed differential abundance between experimental conditions based on the Log standardized protein abundance were required to have a Student *t* test *p*-value ≤ 0.03. (3) Spots should not be observed to overlap with adjoining protein spots on the gel as this could result in contamination with unrelated neighbouring proteins which would result in false positives. Following this filtering, differentially expressed proteins were narrowed down to 22 proteins (Table 1). Of these, 16 were shown to increase in abundance and six were shown to decrease in abundance, in the salt-treated samples compared to the control samples; with 13 of the 22 spots showing fold-changes greater than ± 2 (Table 1). Graphical view of the standardized log abundance of these spots in control vs salt-treated samples from each of the four biological replicates as well as the average standardized log abundance is shown in Additional file 1B. A list of the protein spots fold change relative to control samples and Students *t* test *p*-values are shown in Table 1.

**Table 1:** Results from DIGE analysis of control vs salt treated EBC extract

DIGE									
Spot No.	t-test^a^	Av. Ratio^b^	Protein(s) in Spot^c^	GO location^d^	GO biological process^e^	Uniprot #	Species	Signifcant Unique peptides^f^	M.W./pI^g^
664	0.00013	2.48	V-ATPase A subunit	TP	transport	Q9AVU8	*M. crystallinum*	12	69/5.09
740-1			Nitrite reductase	CP	nitrogen metabolism	B9RYH9	*R. communis*	4	66/6.04
	9.70E-05	2.61							
740-2			Beta-hexosaminidase 1	V	carbohydrate metabolism	A7WM73	*A. thaliana*	3	61/5.88
760	0.00031	2.93	V-ATPase B subunit	TP	transport	Q8GUB5	*M. crystallinum*	31	54/4.96
765	0.0002	2.68	NADP-dependent malic enzyme	Cyt	CAM	P37223	*M. crystallinum*	46	64/6.06
767	0.00062	2.30	NADP-dependent malic enzyme	Cyt	CAM	P37223	*M. crystallinum*	49	64/6.06
774	0.0033	1.67	NADP-dependent malic enzyme	Cyt	CAM	P37223	*M. crystallinum*	48	64/6.06
775	0.0071	1.58	NADP-dependent malic enzyme	Cyt	CAM	P37223	*M. crystallinum*	54	64/6.06
923	0.0016	−1.79	no proteins identified						
1062	0.00053	−1.59	Alpha-1,4-glucan protein synthase	AP	cell wall metabolism	I1J637	*G. max*	16	42/5.52
1064	0.0014	2.00	no proteins identified						
1065	0.0026	1.96	Beta-D-galactosidase	AP	cell wall metabolism	Q5CCQ1	*P. pyrifolia*	3	41/5.5
1071-1			2-phosphoglycerate hydrolase	Cyt	glycolysis	Q43130	*M. crystallinum*	12	48/5.62
	0.001	2.00							
1071-2			Alpha-1,4-glucan protein	Cyt	cell wall metabolism	I1J637	*G. max*	9	42/5.52
1095	0.0034	1.69	Malate dehydrogenase	Cyt	CAM	Q645N1	*S. lycopersicum*	4	36/8.87
1123-1			Malate dehydrogenase	Cyt	CAM/	Q645N1	*S. lycopersicum*	5	36/8.87
	0.00049	2.13							
1123-2			Proline iminopeptidase	Cyt	proteolysis	B9G1Q0	*P. trichocarpa*	4	37/8.96
1153	3.20E-07	6.20	V-ATPase E subunit	TP	transport	Q40272	*M.crystallinum*	26	26/6.52
1290	0.032	−1.43	no proteins identified						
1307	0.0019	2.20	Ascorbate peroxidase	Cyt	stress response	C5J0H6	*S. nigrum*	5	18/4.83
1325	0.0082	−1.66	Fructose bisphosphate aldolase,	Cyt	glycolysis	O04975	M. crystallinum	8	38/6.49
1501	2.40E-07	−2.51	Alpha-galactosidase-like protein	AP	cell wall metabolism	D7LVE6	*A. lyrata*	3	48/4.79
1502	0.003	1.63	5-methyltetrahydropteroyltri-glutamate-homocysteine methyltransferase	Cyt	amino acid metabolism	P93263	*M. crystallinum*	62	85/5.9
1503	0.03	−1.4	Triosephosphate isomerase	Cyt	glycolysis	I3SN66	*M. tribuloides*	5	33/6.54
1508	4.40E-05	2.4	V-ATPase A subunit	TP	transport	Q9AVU8	*M. crystallinum*	54	69/5.09

Data are from 4 independent biological experiments for each treatment^a^Student’s *t*-test *p* values are given as a measure of confidence for the ratio of each spot measured

^b^Average ratios of spot abundance of salt-treated samples relative to the untreated control represent data from four independent experiments

^c^Protein names, ^d^GO cellular location and ^e^GO biological function annotations are taken from the Uniprot database recommended name and annotation

^f^ *p* < 0.05; Peptide sequences, % sequence coverage, best Mascot ion, charge and delta ion score as well as spectra charge states can be seen for all identified proteins in Additional file 5

^g^Theoretical molecular weight and isoelectric point for each protein spot identified

Protein identity following LC-MS/MS (protein threshold 99 %, peptide threshold 95 %, at least two unique peptides) was successfully achieved at high confidence for 19 of the 22 spots (Table 1 and Additional file 2). We were unable to detect protein in three spots (923, 1064, and 1290) and in two of the spots (740 and 1123) we identified more than one protein. These 19 spots corresponded to 14 different proteins. Products expressed from a single gene can migrate to multiple spots on 2D gels for a variety of reasons indicating protein modifications leading to a change in overall protein charge and/or molecular weight (MW) such as splice variants, proteolytic cleavage products, and processed proteins, as well as post-translational modified proteins [13].

In parallel, a complementary proteomics approach was carried out using GeLC–MS/MS, in which protein, from EBC extracts collected from control and salt-treated plants under the exact same conditions as for the 2D-DIGE analysis, was separated by 1D-GE. Each lane, representing one biological replicate of a total of 3, was then sliced into seven pieces as indicated in Additional file 3. This was followed by in-gel digestion and analysis of the resulting tryptic peptide mixtures by LC-MS/MS. In total, 1731 unique peptides derived from 438 proteins were identified in the six EBC samples. For subsequent analyses, only those proteins that were detected in all three biological replicates of either control or salt-treated samples (or both) by at least two unique peptides were considered (225 proteins). For the analysis of the presence/absence of the proteins in different biological replicates, unweighted spectral counts were used.

A quantitative comparison between control and salt-treated EBC samples using spectral counting as a measure of protein abundance and applying several methods including Total spectra count (TSC), Weighted spectra count (WSC), Exponentially Modified Protein Abundance Index (emPAI), and Normalized Spectral Abundance Factor (NSAF) [14, 15] was applied to identify salt-responsive proteins (Table 2). Quantification by spectral count has been shown to be a simple but reliable index for relative protein quantification and has been proven to be both reproducible and accurate over a large dynamic range [15, 16]. To evaluate the significance of comparative quantification by each of the spectral count methods Student’s *t*-test was performed on the data as this has been shown to be most appropriate when comparing three or more replicates as was the case in this study [17], and differences were assigned to be significant at either a *p* value of ≤ 0.05 (*) or a *p* value of ≤ 0.01 (**), and the fold change of abundance of salt-treated to control was selected as greater than ± 2 (Table 2). In total, 40 proteins (approximately 18 %) met the criteria and showed significant changes in salt-treated samples as compared to control, untreated samples. Of these 14 were significant using all four spectral count methods, while 13 and 12 were significant using three or two methods, respectively (Table 2). Only one of the proteins was significant using only a single method. Two of the proteins identified were exclusive to the salt-treated samples and four were only detected in the control samples, showing reported fold changes of either infinity, or zero, respectively (Table 2). We chose not to give these proteins an arbitrary fold-change value as it has been shown that errors in protein ratios can be made when minimum spectral counts in one sample equals zero [18].
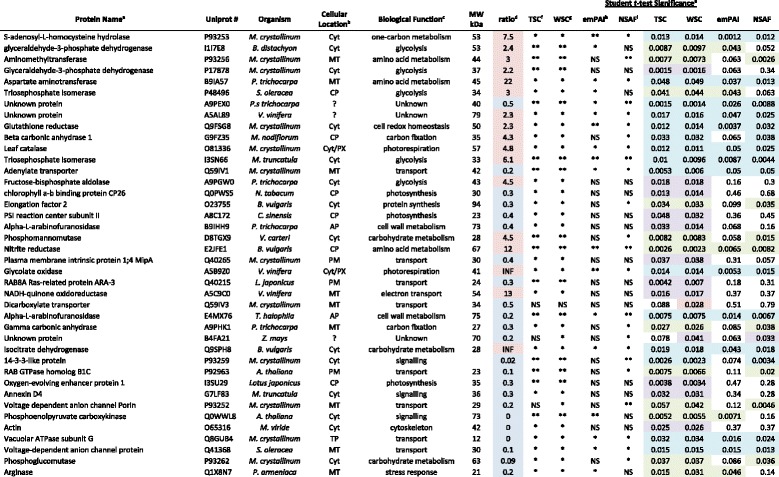

**Table 2:**
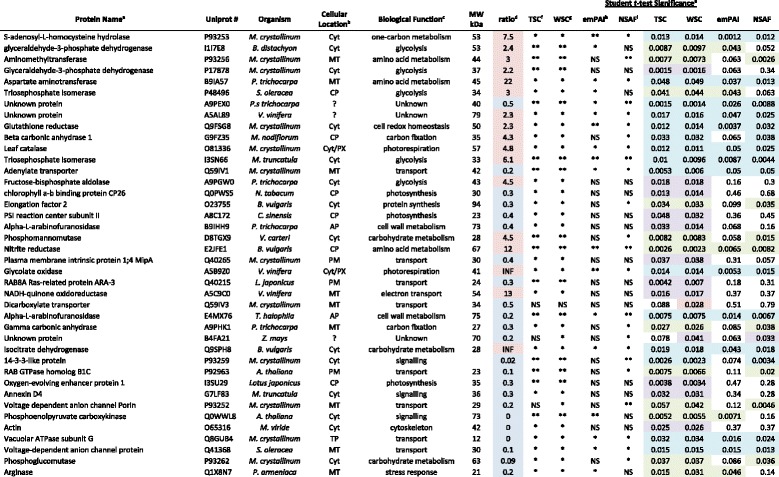
Salt-responsive proteins identified by GeLC-MS Label Free Quantitative Proteomics

^a^Protein names, ^b^GO cellular location and ^c^GO biological function annotations are taken from the Uniprot database recommended name and annotation. Cyt-cytoplasm, MT-mitochondria, CP-chloroplast, PX-peroxisome, AP-apoplast, PM-plasma membrane, TP-tonoplast

^d^Average ratio of abundance of salt-treated spectra relative to the untreated control represent data from three independent experiments. Blue – decrease in abundance, Red – increase in abundance. For those proteins where values are missing (ie. 0), as spectra for the protein were not detected in any biological replicates of the sample, values for fold change will be reported as either 0 or infinity (INF).

^e^Significance of the changes were calculated using Students *t*-test as a measure of confidence for the ratio of each spot measured. ** *p* < 0.01, *p < 0.05, NS - not significant. In the case of total spectral counts (TSC; unweighted spectral counts), the spectras are counted in each of the proteins it is assigned to. For the weighted spectral counts (WSC) the spectra is only assigned to a single protein with the most evidence. Blue highlight indicates that the change was significant using all four methods; Green for three out of four; Purple for two out of four and red for only a single method.

^f^TSC, total spectra count.

^g^WSC, weighted spectra count

^h^emPAI, exponentially modified protein abundance index

^i^NSAF, normalized spectral abundance factor

**Fig. 1:**
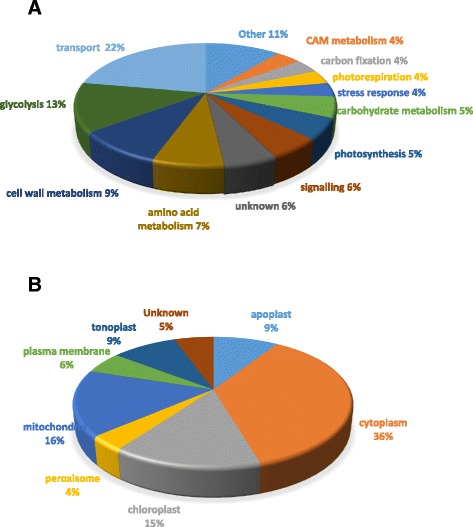
Gene Ontology (GO) term enrichment analysis of identified proteins in the category of (**a**), biological function and (**b**), cellular location. According to UniProtKB GO annotations

Combining the differentially abundant proteins identified in this study (2D-DIGE and GeLC-MS/MS) revealed that the majority of proteins (22 %) could be categorized by GO biological annotation [19] as functioning in transport, including subunits of the tonoplast H^+^-ATPase (V-ATPase) (Fig. 1a). The next most represented category was glycolysis (13 %), followed by cell wall metabolism (9 %) (Fig. 1a). GO cellular location revealed a high proportion of cytoplasmic proteins (36 %), but also proteins from diverse cellular locations including chloroplast and mitochondrial proteins as well as membrane proteins from the plasma membrane and tonoplast (Fig. 1b).

### Western blot validation of proteomics results

**Fig. 2:**
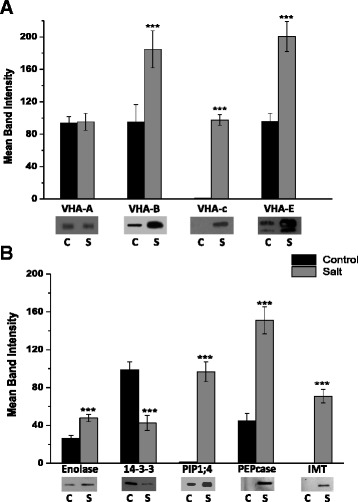
Western blot analysis of EBC extracts for validation of proteomics results. Graphical representation of mean band intensity (*top*) and western blots (*below*) of EBC extract total protein isolated from control (C, *black bars*) and salt-treated (S, *grey bars*) *M. crystallinum* plants. Blots were probed with; (**a**) polyclonal antibodies against the subunits of the V-ATPase (VHA-A, VHA-B, VHA-c and VHA-E), or (**b**) polyclonal antibodies against the glycolytic enzyme enolase, ENO; the 14-3-3 general regulatory factor protein, 14-3-3; the plasma membrane aquaporin, PIP1;4; the CAM enzyme phosphoenolpyruvate carboxylase, PEPC and inositol methyl transferase, IMT. Western blot analysis was carried out as described in the Methods section. Blots are representative of three independent experiments. Unpaired two-tailed Student’s *t*-test (*p* ≤ 0.05) was performed on the mean band intensity data to determine the significance difference

In order to confirm the changes in abundance of proteins from the quantitative proteomic data we selected nine protein candidates for which peptide specific antibodies were available and performed western blot analysis (Fig. 2a and b). Calculation of the average normalized band density from three blots of distinct biological replicates showed significant and complementary changes in amounts of the majority of detected proteins selected. However, two exceptions were noted. The first being VHA-A, which showed no significant change in protein amount on the western blot (Fig. 2a), despite showing a 2.5-fold change in abundance in the DIGE Decyder analysis (Table 1). The other exception was the aquaporin PIP1;4, which showed a significant increase in the western blot analysis (Fig. 2b), but was identified as a down-regulated protein in the proteomic analysis (Table 2).

### EBC chloroplasts

The majority of epidermal cell types do not contain chloroplasts, with the exception of the guard cells [20], and some trichomes [21]. Although early work raised doubt about the presence or functionality of chloroplasts in *M. crystallinum* EBC [10], more recent studies relying on new technologies including proteomics and single cell-type sampling methods have identified proteins associated with photosynthesis [9, 22].

**Fig. 3:**
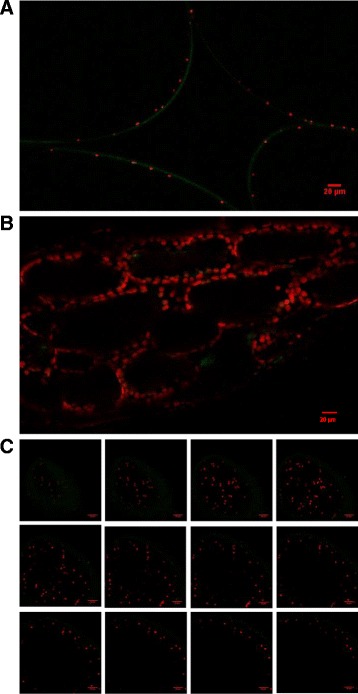
Visualization of EBC chloroplast autofluorescence by confocal laser scanning microscopy. Stem sections from salt-treated plants were submerged in water and images were obtained using an Olympus FV1000 confocal laser scanning microscope using an XLPLN 25X W NA:1.05 water immersion objective lens. Laser wavelength 1 = 488 (*green*) cell wall autofluorescence, Laser wavelength 2 = 635 (*red*) chloroplast autofluorescence. (**a**) Chloroplasts in EBC and (**b**), mesophyll cells. (**c**) Each panel is a single confocal section taken from a Z-stack of 12 confocal images acquired at 20 μm intervals

Many of the chloroplast proteins identified in this study from GeLC-MS/MS analysis of either control or salt-treated EBC samples, including a number which are salt responsive, are components of the two photosystems (Tables 2). In order to confirm the presence of photosynthetically active chloroplasts in the EBC, confocal scanning microscopy was used to detect chlorophyll auto fluorescence. Red intrinsic fluorescence by chloroplasts indicates photochemical activity and specifically is a measure of photosystem II activity [23]. As observed in Fig. 3a, red fluorescence from chloroplasts was seen around the periphery of the large EBCs (delineated by green autofluorescence of the cell wall), and these chloroplasts are clearly distinguishable from the chloroplasts in the underlying mesophyll cell layer (Fig. 3b). Z-sequences of optical slices from the top of the EBC down towards the base of the EBC highlights the abundance and distribution of the chloroplasts in the bladder cells (Fig. 3c).

### pH and Malate concentration in EBC extract

**Fig. 4:**
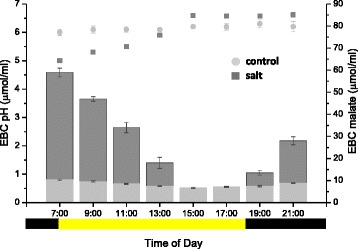
Measurement of pH and malate in EBC extract. Malate (*bar graphs*) and pH (*scatter plot*) were measured in leaves of control (*light grey bars and symbols*) and salt-treated (*dark grey bars and symbols*) plants at 2 h intervals from 7:00 to 21:00. The yellow horizontal bar represents the light period and black bars represent the dark period. Values are expressed as means of three independent experiments with standard deviation (SD) shown not exceeding 10 %

Our RNAseq analysis and this proteomics study identified transcripts and proteins for multiple essential CAM genes, with many showing significant salt induction [24] (Tables 1 and 2), suggesting that EBCs may contribute to the carbon fixation process in the leaf. To verify this we followed the diurnal fluctuations in pH and organic acids in the EBC extract. EBC from salt-treated plants showed the classic day/night changes in pH characteristic of CAM which were absent in the untreated control plants (Fig. 4). These changes were directly related to the nocturnal storage of CO_2_ in the form of organic acids and the subsequent remobilization and decarboxylation of the organic acids during the light period [25]. As malate is the predominant form of organic acid stored in most CAM plants [26], the malate content of the EBC was measured from the end of the dark period to the end of the light period (Fig. 4). Diurnal fluctuations in malate content in salt treated plants correlated closely with the changes in pH measured in EBC, however at the end of the light period there was a more rapid reduction in malate levels than was reflected by changes in the pH. This suggested the presence of additional organic acids such as citrate in the EBC, whose decarboxylation during the day has been shown to be delayed in comparison to malate in species undergoing CAM metabolism [27]. Metabolomic profiling of EBC extracts at the start of the light period has shown the presence of other dicarboxylic acids, however only maleate, a trans-isomer of fumaric acid, was shown to be significantly altered following salt treatment at this time of day [28]. No diurnal fluctuations were observed in malate levels in control untreated plants and pH remained stable over the day/night cycle (Fig. 4).

### Ionomics

**Fig. 5:**
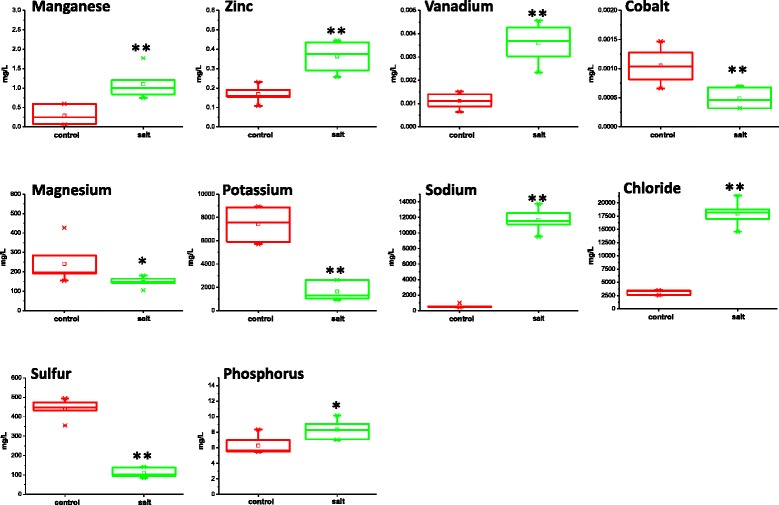
Box plots of the significant changes in ion concentration of EBC from control and salt-treated plants. For each concentration, the box represents the interquartile range (IQR), the bisecting line represents the median, the square symbol represents the mean, the whiskers represent the 95th and 5th percentiles, and the X symbols represent the maximum and minimum values. Elements which are significantly different between treatments with a *P* < .01 are designated **, while those significantly different with *P* < 0.05 are designated *

The combined use of ICP-MS (Inductively Coupled Plasma - Mass Spectrometry) or ICP-OES (Inductively Coupled Plasma - Optical Emission Spectrometry) enabled the determination of 27 major and trace elements (Al, As, Ba, B, Br, Cd, Ca, Cl, Cr, Co, Cu, Fe, Pb, Hg, Mg, Mn, Mo, Ni, P, K, Se, Si, Ag, Na, S, V, and Zn) in EBC extracts with high accuracy and precision. The elemental concentrations varied by almost seven orders of magnitude (Additional file 4). The least abundant elements measured in the EBC extract from both control and salt-treated plants were Cd, Cr, Pb, Hg, V, Co, all of which were below 0.05 mg/L. The most abundant element in the EBC extract from control plants was K, while the most abundant elements in the EBC extract from salt-treated plants was Cl followed by Na (Additional file 4). Salt-treatment strongly affected the accumulation of numerous elements (Fig. 5). As expected, and has been shown previously [8, 11, 24], Na increased significantly in the EBC (21-fold), from an average of 557 mg/L in control samples to 11,679 mg/L in the samples from salt-treated plants. Cl also showed a large increase of 5.7-fold; from 3144 mg/L in the control extract to 18,000 mg/L in the salt-treated extract. Significant increases were also seen in the abundance of Mn, P, V and Zn (Fig. 5). Elements that showed a significant decrease in abundance relative to control in the samples from salt-treated EBC were K, S, Mg and Co (Fig. 5) with K decreasing 4.5-fold from an average of 7429 mg/L in the EBC from control plants to only 1637 mg/L in salt-treated plants and sulphur decreasing 4-fold from an average of 442 mg/L in control plants to 111 mg/L in salt-treated plants (Additional file 4 and Fig. 5).

**Fig. 6:**
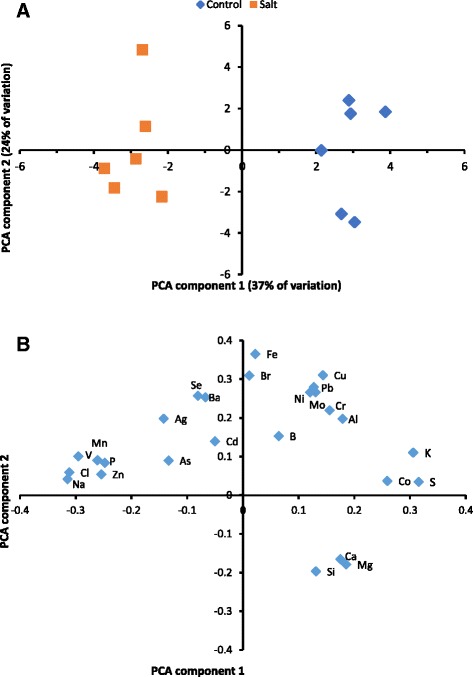
Results from principal components analysis (PCA) of mineral data with (**a**) scores for each plant and (**b**) loadings for each element plotted for the first two PCA components

Multivariate data processing and study of element distribution patterns provided additional information about the ionomic response of the EBC to salt-stress. Principal Component Analysis (PCA) of the correlation matrix, with the element concentrations regarded as variables and the samples as objects, indicated a good separation of the PCA scores for the control and salt-treated plants (Fig. 6a). The first two PCA components explained 37 and 24 % of the variation, respectively, and the main discrimination between control vs salt plants was along component 1. The loadings plot (Fig. 6b) indicates that the key elements discriminating control from salt-treated plants were K, S and Co (which were positively loaded on the first component) whilst Na, Cl, V, Mn, P and Zn all had negative loadings.

## Discussion

The development of a high throughput and precise technique to sample the extract of the epidermal bladder cells from *Mesembryanthemum crystallinum* provides a unique opportunity to understand function and regulation of genes and pathways with concise contextual information from a single cell type. In combination with multi-omics approaches, we can begin to build a comprehensive integrated picture of cellular processes within the bladder cell. In this study, we performed both label and label-free proteomics and carried out ionomics analysis of the EBC extracts from control and salt-treated plants, to complete our omics analysis of these cells [24, 28].

The complementary nature of the quantitative proteomics technologies used in this study (2D-DIGE and 1D-GE label free) is underscored by the lack of overlap in the proteins identified (Tables 1 and 2). This highlights the advantage of combining different approaches and techniques to obtain a greater coverage of the proteome. Differences in sample handling, from the composition of the sample buffers to the gel separation conditions, combined with the physicochemical properties of the proteins in the sample result in unique differences in protein profiling between the two approaches helping to maximize the number of proteins identified. Using GeLC-MS/MS we were able to identify 141 more proteins than had previously been identified employing shotgun LC-MS/MS; an increase of 2.7 fold [9]. These numbers are based on the identification of at least two unique peptides and the protein being present in all three biological replicates of either control or salt-treated samples for GeLC-MS/MS (this study), or two of four biological replicates from our previous LC-MS/MS profiling study [9]. Using this criteria, only 11 proteins were exclusive to the previous LC-MS/MS analysis. Obtaining comprehensive protein profiles from very complex samples is challenging due to the large number of proteins present in the sample over a wide dynamic range in abundance. In the EBC extract a cysteine protease makes up nearly 50 % of the identified spectra in the samples [9], and is the most abundant protein on SDS-PAGE gels (Additional file 3 - asterisks). The high abundance of this protein would result in an under sampling of the low abundant proteins in the fraction. GeLC-MS/MS helped to overcome this problem by decreasing sample complexity, and when directly compared to LC-MS/MS in this study and others [29, 30], it was shown to perform better in the number of protein identifications, reproducibility of identifications and % coefficient of variance on spectral counts.

**Fig. 7:**
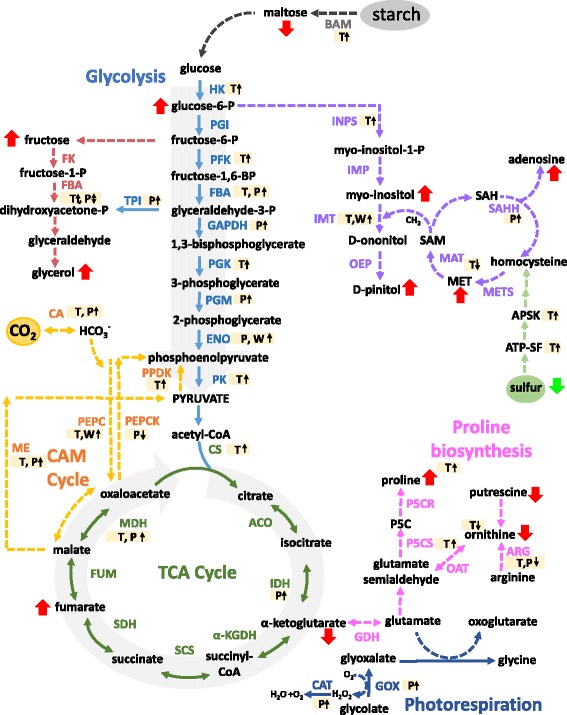
Integration of transcript, protein, metabolite and ionome data into EBC metabolic pathways. Data from all studies was obtained from 6-week-old plants treated for 2 weeks with 200 mM NaCl. Bladder cell extract was collected at the end of the dark period. Transcriptomic data from Oh et al., [24] and metabolomics data from Barkla and Vera-Estrella, [28]. T, transcript; P, protein and W, western blot analysis. Red arrows indicate changes in metabolites. Enzyme abbreviations: BAM – ϐ-amylase, HK – hexokinase, PGI – glucose-6P-isomerase, PFK – phosphofructokinase, FBA – aldolase, TPI – triose-P-isomerase, G3PD – glyceraldehyde-3P-dehydrogenase, PGK – phosphoglycerate kinase, PGM – phosphoglycerate mutase, ENO – enolase, PK- pyruvate kinase, FK – fructokinase, CS – citrate synthase, ACO – aconitase, IDH – isocitrate dehydrogenase, α-KGDH - α-ketoglutarate dehydrogenase, SCS – succinyl-CoA synthetase, SDH – succinate dehydrogenase, FUM – fumerase, MDH – malate dehydrogenase, PEPCK – PEP carboxykinase, ME – malic enzyme, PEPC – phosphoenolpyruvate carboxylase, PPDK – pyruvate-Pi-dikinase, CA – carbonic anhydrase, GDH – glutamate dehydrogenase, P5CS - pyrroline-5-carboxylate synthase, P5CR - pyrroline-5-carboxylase reductase, OAT - ornithine aminotransferase, ARG – arginase, INSP - myo-inositol 1-phosphate synthase, IMP – myo-inositol monophosphatase, IMT – inositol methyl transferase, OEP - ononitol epimerase, MAT – methionine adenosyltransferase, SAM - S-adenosyl methionine, SAH - S-adenosylhomocysteine, SAHH – S-adenosylhomocysteine hydrolase, MET – methionine, METS – methionine synthase, ATP-SF - ATP-sulfurylase, APSK - 5’-adenylylsulfate kinase, GOX - glucose oxidase, CAT – catalase

**Fig. 8:**
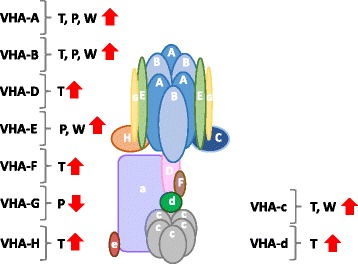
Integrated analysis of changes in transcript and protein abundance in EBC V-ATPase subunits. V-ATPase structure was adapted from Forgac, [57]. Changes in transcript abundance were taken from Oh et al., [24]. T, transcript; P, protein and W, western blot analysis

Multi-omics data integration is challenging for plant-derived pathways and particularly for non-model plants, however, better insight into functional networks can be gained if we incorporate data compiled from different technologies. From information from this study and our previous omics analysis of EBC [24, 28], a picture is emerging of a metabolically active cell, photosynthetically active and undertaking CAM, with salinity treatment resulting in decreased abundance of photosynthetic machinery proteins, increases in enzymes involved in photorespiration, glycolysis and proteins specific for CAM. (Fig. 7). High metabolic activity highlights the considerable energy cost to drive compatible solute synthesis and Na accumulation in the cell [31]. From the 54 proteins that showed significant fold changes with salt treatment and were present in all biological replicates, a high proportion of those identified can be classified as transport proteins (Fig. 1a). Of these, four were subunits of the peripheral cytoplasmic V_1_ sector of the vacuolar H^+^-ATPase, V-ATPase; VHA-G, VHA-A, VHA-B and VHA-E (Fig. 8). Western blot analysis using subunit specific antibodies confirmed the increase in VHA-B and VHA-E, (Fig. 2) and results were corroborated for VHA-A and VHA-B from our previous EBC transcriptomics study [24]. Transcriptomics data also revealed changes in additional VHA V1 subunits, including VHA-D, VHA-F, VHA-H, but also subunits of the V_0_ membrane sector, VHA-c and VHA-d (Fig. 8). While these were not detected in the proteomics study, western blot analysis was able to confirm the change in abundance of VHA-c (Fig. 2). These results for EBC extracted total protein are in agreement to results reported for whole leaf microsomal proteomic analysis of *M. crystallinum* [32]. In that study significant changes in subunits VHA-A, VHA-E, but also VHA-a, were identified; additionally, although not significant, changes in relative abundance were also measured for VHA-B, VHA-G, VHA-H, VHA-c and VHA-d [32]. However, results would comprise a mix of protein originating from up to 15 cell types with diverse functions in the leaf [33]. In plants, the V-ATPase is not only present on the vacuolar membrane (tonoplast) but also endosomal vesicular compartments, where it has a role in luminal pH control, vesicle trafficking and generation of an electrochemical gradient for ion transport. Recent evidence employing V-ATPase mutants showed that the tonoplast localized V-ATPase does not play a role in salt tolerance, as despite lacking a functional tonoplast V-ATPase, mutants were still able to accumulate sodium [34]. Moreover, although capturing multiple full-length transcripts for tonoplast localized Na/H exchangers (NXH), none of them showed significant induction in response to salt [24], and no NHX proteins were identified in our quantitative proteomics analysis (Tables 1 and 2). Therefore, changes in abundance of V-ATPase in EBC observed in this study may be important for energizing the uptake of sodium into endosomal vesicles [35, 36], which are then delivered to and fuse with the tonoplast. Additionally, V-ATPase activity would be essential for turgor generation to facilitate rapid cell expansion of the EBC [37]. The role of the V-ATPase in determining cell shape and size through turgor generations has been demonstrated by studying Arabidopsis VHA-C mutants, which showed reduced cell expansion of specific cell types due to reduced turgor [38].

Ionomics analysis of elements reveals that the EBC Na/K ratio goes from 0.075 mg/L in the EBC from control plants to 7.133 mg/L in the EBC from salt-treated plants, a 100-fold difference (Additional file 4). Salinity commonly reduces the amount of K in cells from both glycophytes and halophytes [39]; however, in halophytes Na can substitute for K for turgor generation and cell growth [40].

The combined accumulation of Na and K usually exceeds Cl by about 35 % in dicotyledonous species and by at least double in halophytic grasses [39]. In this study, while control untreated plants had a combined accumulation of Na and K more than double that of the Cl content (Cl_av_ = 3144 mg/L; Na_av_ + K_av_ = 7985 mg/L), in the salt treated plants the Cl content was 1.4 fold that of Na_av_ + K_av_ (Additional file 4). Chloride was the most accumulated ion, exceeding Na by 1.4 fold, suggesting an important role of EBC in Cl accumulation and detoxificaiton. Few studies of salt tolerance traits have linked tolerance to chloride homeostasis, and mechanisms of chloride transport into and within cells is poorly understood in comparison to Na transport [41]. Of two possible chloride channels belonging to the CLC family of anion transporters only one, CLC-b, which is thought to be tonoplast localized from studies in Arabidopsis [42], was found to be significantly upregulated in our RNA-seq analysis of EBC [24].

Significant increases in the levels of manganese were also detected in the salt-treated plants (Fig. 5). This micronutrient activates decarboxylase, dehydrogenase and oxidase enzymes and is therefore an essential regulator for both glycolysis and CAM enzymes [43]. Additionally, Mn is important for redox systems, as activators of various enzymes including those involved in the detoxification of superoxide radicals [44] and therefore increases may be linked to stress-induced ROS production.

## Conclusions

The view of the EBC as a simple passive storage body for sodium and water is mistaken. Rather, our single-cell-type omics approach, combining proteomics and ionomics in this study, with transcriptomics [24], and metabolomics data [28], shows indisputably that these specialized cells are highly metabolically active, with photosynthesis and primary metabolism supporting rapid cell expansion, ion accumulation, compatible solute synthesis and CAM.

## Methods

### Plant materials and growth conditions

*Mesembryanthemum crystallinum* L. plants were germinated in potting substrate (MetroMix 510; SunGro Horticulture, Bellevue, WA) in a propagation tray. Three weeks following germination, individual seedlings were transplanted to pots containing potting substrate at a density of two plants per 15-cm-diameter pot. The watering regime consisted of daily watering with tap water with a weekly supply of Hoagland’s medium [45] until plants were 6 weeks old. At that time NaCl treatment (200 mM) was initiated for a period of 14 d with either water (for control plants) or NaCl (for salt-treated plants) supplied daily. Plants were grown in a glasshouse under natural irradiation and photoperiod at, 18.93 latitude and -99.23 longitude and an elevation 1540 m above sea level in the months of March to June. Temperature was maintained at 25 °C ± 3 °C and peak photosynthetic photon flux density was 1300 mmol m^−2^ s^−1^ during the middle of the day.

### Extraction of bladder cell Sap

Vacuum aspiration was applied to collect bladder cell sap from individual cells on the leaf or stem epidermal surface using a fine gage insulin needle (27G, 13 mm) attached to a collection reservoir maintained on ice. To avoid contamination of cellular contents from underlying cell types the collection needle was oriented horizontally to the leaf or stem axis and the procedure was visualized using a Nikon SMZ645 stereo microscope equipped with a dual arm Nikon MKII fibre optic light source (Nikon, Japan). Extracts for a single plant were pooled to obtain approximately 1 mL of sample (approximately 3000 EBC), representing a single biological replicate and in this way distinct biological replicates as indicated for the individual experiments were collected (2D-DIGE – 4 biological replicates and Label-free Proteomics – 3 biological replicates).

### Protein determination in samples

Protein in EBC extracts was measured by a modification of the Bradford method [46]. Triton X-100 [0.5 % (v/v)] was added for 5 min before dilution of the sample and the addition of the dye reagent concentrate (Bio-Rad); the final concentration of Triton X-100 in the assay following dilution was 0.015 % (v/v). Protein in samples prepared for 2D-DIGE analysis was measured by the RCDC Protein Assay Kit (Bio-Rad) according to manufacturer’s instructions. For both methods BSA was employed as the protein standard.

### 2D-DIGE Analysis

EBC sap was diluted in 2X concentrated TE buffer (final; 10 mM Tris/HCl pH 7.6; 1 mM EDTA pH 8; 0.1 % (w/v) sodium deoxycholate) and samples were precipitated sequentially; first with 72 % (w/v) TCA, followed by 90 % (v/v) acetone. Protein (75 μg) was then desalted/cleaned according to manufacturer’s instructions with the ReadyPrep 2D Cleanup kit (Bio-Rad). The final protein pellet was resuspended in labelling buffer; 30 mM Tris-HCl pH 8.5, 7 M urea, 2 M thiourea, 2 % CHAPS (w/v), 2 % (w/v) amidosulfobetaine-14 (ASB-14). Samples were then labelled with the appropriate CyDye (Cy2, Cy3, or Cy5) according to the strategy outlined in the experimental design (Additional file 5). To each sample, 300 pmol of the appropriate dye was added and samples were incubated for 30 min on ice in the dark. The labelling reaction was stopped by the addition of one μl of 10 mM lysine and incubated on ice for a further 10 min. To avoid CyDye specific artifacts resulting from preferential labelling or variable fluorescence characteristics of the gel matrix or glass plates at the different excitation wavelengths used for acquisition, dye swapping between experimental samples was carried out (Additional file 5). Following labelling, equal volumes of rehydration buffer containing 2X DTT and ampholytes was added to each sample (7 M urea, 2 M thiourea, 2 % (w/v) ASB-14, 2 % (w/v) CHAPS, 100 mM DTT, 1 % (v/v) Bio-Lyte 3–10 ampholytes (Bio-Rad) to give a final concentration of 50 mM DTT and 0.5 % (v/v) Bio-Lyte 3–10 ampholytes. The three different CyDye labelled samples for each gel were then pooled and brought to a final volume of 300 μl with rehydration buffer containing 50 mM DTT and 0.5 % ampholytes.

### 2D Gel electrophoresis, Gel imaging and image analysis

For rehydration Ready Strip IPG strips (17 cm, linear pH 3–10, Bio-Rad) were layered gel side down onto CyDye labelled samples placed in the Protean IEF tray (Bio-Rad) ensuring bubbles were not trapped under the strip. Strips were carefully covered with 2 ml of mineral oil and active rehydration was carried out overnight in a Protean IEF Cell (Bio-Rad) at 50 V and 20 °C in the dark. Following overnight rehydration of the strips, isoelectric focusing (IEF) was initiated for a total of 40,000 volt hours with a maximum current setting of 50 μA per strip, using a three-step ramping protocol. After IEF the IPG strips were first equilibrated by shaking for 15 min in DTT equilibration buffer (6 M urea, 0.375 M Tris-HCl pH 8.8, 2 % (w/v) SDS, 20 % (w/v) glycerol and 2 % (w/v) DTT), and then for an additional 15 min in iodoacetamide equilibration buffer (6 M urea, 0.375 M Tris-HCl pH 8.8, 2 % (w/v) SDS, 20 % (w/v) glycerol and 2.5 % (w/v) iodoacetamide). Equilibrated gel strips were loaded onto 10 % acrylamide gels cast between low fluorescence glass plates coated on one side with bind silane solution (80 % (v/v) ethanol, 2 % (v/v) glacial acetic acid and 0.001 % (v/v) bind silane). Two fluorescent reference markers were included on opposite sides of the glass plate containing bind silane to facilitate robotic spot picking. Strips were overlaid with 0.5 % (w/v) low melting point agarose in SDS running buffer (25 mM Tris-HCl, pH 8.3, 192 mM glycine, 0.1 % (w/v) SDS and 0.01 % (w/v) Bromophenol Blue) and SDS-PAGE was carried out using the Ettan Daltsix electrophoresis system (GE Lifesciences) at 10 mA/gel for 1 h followed by 12 mA/gel for a total of 17–20 h, in the dark at 25 °C.

Individual gels were scanned at three different wavelengths using a Typhoon Variable Mode TM 9410 imager (GE Lifesciences) to obtain the images for each of the three CyDyes according to the acquisition conditions outlined in Additional file 6 online. Image analysis was carried out using the DeCyder 2D Software V6.5 following the manufacturer’s instructions (GE Lifesciences) and as described [47].

### Spot picking and protein identification by ESI-LTQ-orbitrap MS/MS

Protein spots of interest were excised from the gels using the Ettan Spot Picker robot (GE Lifesciences) according to the spot pick map generated by the Decyder software for significantly altered spots. Protein spots were sent by overnight courier to the Proteomics Facility at the Institut de Recherches Clinique de Montreal, Canada for processing and MS analysis. Digestion of protein was carried out according to the in-gel method [48]. Protein digests were desalted by solid-phase extraction employing C18-ZipTips from Millipore. Peptides were bound, washed, and then eluted in 15 μl of 1 % (v/v) formic acid in 50 % (v/v) ACN.

The resulting peptide mixtures from each excised spot were analysed by nano LC-MS/MS using a Finnigan MicroAS autosampler and a Surveyor MS pump system coupled to an LTQ-Orbitrap (ThermoFisher Scientific). Forty μL of each peptide mixture was loaded on a C18 precolumn (Symmetry300 C18 5 μm, NanoEase Trap Column, Waters) at 3 μl/min for 15 min in 0.1 % (v/v) formic acid in 5 % (v/v) ACN. Peptides were eluted using a 5–35 % gradient of solvent B (0.1 % (v/v) formic acid in 100 % (v/v) ACN) during 60 min at a flow rate of 300 nl/min with a BioBasic C18 picofrit column (PFC7515/BI/10, NewObjective). Data-dependent acquisition mode was carried out with the Xcalibur software. A Fourier transformed (FT) full scan from 300 to 1800 m/z was acquired by means of the Orbitrap, with resolving power set at 30,000 (400 m/z). The five most intense peaks were sequentially isolated for the MS/MS experiments using collisionally induced dissociation. Dynamic exclusion was set to two and selected ions were placed on the exclusion list for 45 s to prevent duplication of MS/MS data for the same peptide. The MS/MS raw spectra data were converted to DTA files using ThermoElectron Bioworks 3.2 and analyzed by means of Turbo SEQUEST (ThermoFisher Scientific). From a general Viridiplantae_txid33090 database (unknown version, 677107 entries) two decoy databases were generated for *M. crystallinum* and *A. thaliana*. Independent searching was carried out using rigorous parameters (Xcorr z = 1: 1.90, z = 2: 2.70, z = 3: 3.50, z = 4: 3.75 and Delta Cn >0.1) and allowing dynamic modifications for cysteine alkylation with iodoacetamide and methionine oxidation.

### Label-free quantitative proteomics

EBC extracts (20 μg protein per sample) were precipitated using 1:1 volumes of ethanol/acetone, resuspended in 2.5 % (w/v) SDS Tris/glycine sample buffer, heated at 60 °C for 2 min, and loaded onto a 10 % (w/v) acrylamide mini-gel. Following electrophoresis (200 V for 55 min) gels were stained in Coomassie Blue and each replicate lane was subsequently sliced into seven pieces as indicated in Additional file 3. Gel slices were processed as described above for gel spots. Data from all gel slices representing a single lane or biological replicate were combined for further analysis.

### SDS PAGE, staining and western immuno-blotting

Protein samples were precipitated by dilution of the samples 50 fold in 1:1 (v/v) ethanol/acetone and incubated overnight at 30 °C according to the method of Parry et al., [49]. Samples were then centrifuged at 13 000 *g* for 20 min at 4 °C using an F2402 rotor in a GS15R table top centrifuge (Beckman). Pellets were air dried, re-suspended with sample buffer (2.5 % (w/v) SDS), and heated at 60 °C for 2 min before loading (15 μg of protein per lane) onto 10 % (w/v) linear mini gels (Bio-Rad). After electrophoresis, SDS-PAGE separated proteins were either fixed and stained with Coomassie R250, or electrophoretically transferred onto nitrocellulose membranes (ECL, GE Lifesciences) for western immunoblot analysis as previously described [50]. Digital chemiluminescent images were captured using a C-DiGit Blot scanner (LICOR Biosciences). Primary antibodies used in this study were either commercially available or custom made as indicated. Antibodies purchased from Agrisera (Agrisera, Sweden) included the *A. thaliana* V-ATPase subunits VHA-A (69 kD; AgriSera Cat# AS09 467 RRID:AB_1832048), VHA-B (55 kD; AgriSera Cat# AS09 503 RRID:AB_1832050), VHA-c (16 kD; AgriSera Cat# AS09 468 RRID:AB_1832051) and VHA-E (29 kD; AgriSera Cat# AS07 213 RRID:AB_1031583); and the general regulatory element 14-3-3 protein GRF (20 kD; AgriSera Cat# AS12 2119). Anti-enolase antibodies were purchased from Santa Cruz Biotechnology (50 kD; cat. #sc7455 AB_640163). Custom and in-house made primary antibodies used included; the *M. crystallinum* phospho*enol* pyruvate carboxylase anti-PEPCase CAM isoform (110 kD) [51]; the *M. crystallinum* aquaporin PIP1;4 peptide specific antibody (41 kD) [52]; *M. crystallinum* myo-inositol O-methyl transferase anti-IMT antibodies (40 kD) [53, 54]. Dilutions were as follows: VHA-A, VHA-B, VHA-c, were 1/2000; all others were 1/1000.

### Ionomics analysis

For analysis of elements, EBC extracts (six biological replicates for each condition) were analysed on a Perkin Elmer NexION 300D ICPMS. The instrument was calibrated for each element using a three-point calibration curve, prepared from certified stock solutions, to provide an R^2^ coefficient of 0.9999 or greater. The accuracy of the calibration was confirmed by analysing standards that are independent from those of the calibrating solution. Calibration standards were re-analysed every 20 samples to confirm calibration stability and suitable internal standards were used to monitor and correct for instrument drift. Polyatomic interferences were removed using helium gas in Kinetic Energy Discrimination (KED) mode, additionally methane was used in Dynamic Reaction Cell (DRC) to remove interferences on selenium. Ionomics data was checked for outliers (>3 SD from the mean) and one value was removed for each of nickel, lead, zinc, silicon and barium for all subsequent analyses. One-way analyses of variance and principal component analysis (PCA) were done using Genstat software [55]. The PCA was based on the correlation matrix to avoid biasing the results towards trait with high variance. PCA loadings and scores were extracted and the values for components 1 vs 2 plotted.

### pH and Malate measurements

The pH of the EBC extract was measured directly in the fluid obtained with a pH micro-electrode PerpHecT Ross microcombination pH electrode (8220BNWP model, Thermo-Fisher Scientific) connected to a pH meter (Accumet, Fisher Scientific).

Malate was quantified enzymatically by a coupled enzyme assay according to Hohorst [56]. The reaction medium contained 50 mM glycylglycine (pH 10), 30 mM L-glutamate, 3 mM NAD+, I U of glutamate oxaloacetate transaminase (GOT, Sigma-Aldrich), 10 U of L-malate dehydrogenase (MDH, Sigma-Aldrich). Malate concentrations were obtained by calculating the difference in the absorbance at 340 nm before and after 20 min incubation at RT. Measurements of pH and malate were made on four independent samples for each time and treatment, and the results for malate were expressed as μmol malate ml^−1^ of EBC extract.

### Confocal microscopy

Fluorescence microscopy was performed using an upright multiphotonic confocal microscope (Olympus FV1000) equipped with an XLPLN 25X W NA1.05 water immersion objective. Stem sections from salt-treated plants were submerged in water for imaging. Laser wavelength 1 = 488 (green) cell wall autofluorescence, Laser wavelength 2 = 635 (red) chloroplast autofluorescence. Chlorophyll autofluorescence was visualized by excitation with a multi-line Argon laser at 635 nm and spectral detector set between 650–750 nm for the emission.

### Availability of data and materials section

The mass spectrometry proteomics data have been deposited to the ProteomeXchange Consortium (http://proteomecentral.proteomexchange.org) via the PRIDE partner repository with the dataset identifier PXD004045.

## Additional files

Additional file 1: Figure showing in A, a representative multiplexed 2D gel of experimental samples used for DIGE and B, graphical representation of spot ratios of significantly altered spots. (PDF 419 kb) Additional file 2: Table of additional protein and peptide information from MS analysis of DIGE spots. (PDF 585 kb) Additional file 3: Figure showing the 1D-SDS-PAGE separated total proteins isolated from EBC used for GeLC-MS/MS. (PDF 263 kb) Additional file 4: Table of the ionomics data to quantify elements in the EBC extract from control and salt-treated plants. (PDF 343 kb) Additional file 5: Table listing the experimental design for the 2D-DIGE analysis. (PDF 96 kb) Additional file 6: Table listing the gel scanning parameters used for the Typhoon Imager. (PDF 7 kb)

## Abbreviations

2D-DIGE: two dimensional differential in-gel electrophoresis
ACN: acetonitrile
ACO: aconitase
APSK: 5’-adenylylsulfate kinase
ARG: arginase
ATP-SF: ATP-sulfurylase
BAM: ϐ-amylase
BICAM: ammonium bicarbonate
BVA: biological variation analysis
CA: carbonic anhydrase
CAM: crassulacean acid metabolism
CAT: catalase
CS: citrate synthase
EBC: epidermal bladder cell
emPAI: exponentially modified protein abundance
ENO: enolase
FBA: aldolase
FK: fructokinase
FUM: fumerase
G3PD: glyceraldehyde-3P-dehydrogenase
GDH: glutamate dehydrogenase
GeLC-MS/MS: in-gel tryptic digestion followed by liquid chromatography-tandem mass spectrometry
GO: gene ontology
GOX: glucose oxidase
HK: hexokinase
ICP-MS: inductively coupled plasma - mass spectrometry
ICP-OES: inductively coupled plasma - optical emission spectrometry
IDH: isocitrate dehydrogenase
IEF: isoelectric focusing
IMP: myo-inositol monophosphatase
IMT: inositol methyl transferase
INSP: myo-inositol 1-phosphate synthase
LC-MS/MS: Liquid chromatography-tandem mass spectrometry
MAT: methionine adenosyltransferase
MDH: malate dehydrogenase
ME: malic enzyme
MET: methionine
METS: methionine synthase
MW: molecular weight
NSAF: normalized spectral abundance factor
OAT: ornithine aminotransferase
OEP: ononitol epimerase
P5CR: pyrroline-5-carboxylase reductase
P5CS: pyrroline-5-carboxylate synthase
PCA: principal component analysis
PEPC: phosphoenolpyruvate carboxylase
PEPCK: PEP carboxykinase
PFK: phosphofructokinase
PGI: glucose-6P-isomerase
PGK: phosphoglycerate kinase
PGM: phosphoglycerate mutase
PK: pyruvate kinase
PPDK: pyruvate-Pi-dikinase
SAH: S-adenosylhomocysteine
SAHH: S-adenosylhomocysteine hydrolase
SAM: S-adenosyl methionine
SCS: succinyl-CoA synthetase
SDH: succinate dehydrogenase
SDS-PAGE: sodium dodecyl sulfate polyacrylamide gel electrophoresis
TPI: triose-P-isomerase
TSC: total spectra count
V-ATPase: vacuolar H^+^-ATPase
WSC: weighted spectra count
α-KGDH: α-ketoglutarate dehydrogenase
