# Supplementary material for: Single-cell-type quantitative proteomic and ionomic analysis of epidermal bladder cells from the halophyte model plant Mesembryanthemum crystallinum to identify salt-responsive proteins
Source: BMC Plant Biol. 2016 May 10;16:110. doi: 10.1186/s12870-016-0797-1 (PMC4862212; doi:10.1186/s12870-016-0797-1)
Supplement: Additional file 1: — Figure showing in A, a representative multiplexed 2D gel of experimental samples used for DIGE and B, graphical representation of spot ratios of significantly altered spots. (PDF 419 kb) [file 12870_2016_797_MOESM1_ESM.pdf]

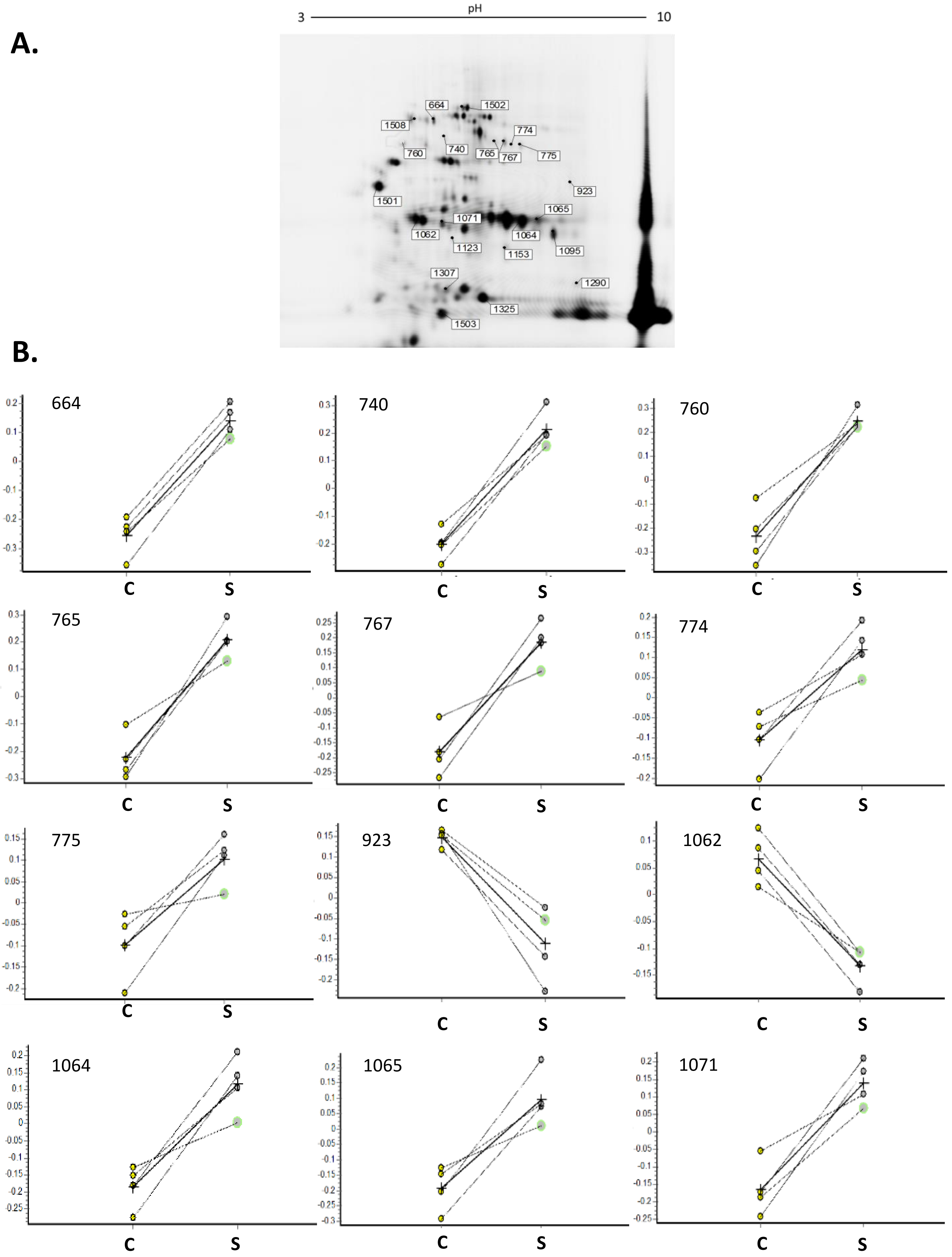

A.
3
pH
10
664
1502
1508
774
760
740
765
767
775
923
1501
1065
1071
1062
1064
1123
1153
1095
1307
1290
1325
1503
B.
664
740
760
765
767
774
775
923
1062
1064
1065
1071
C
S

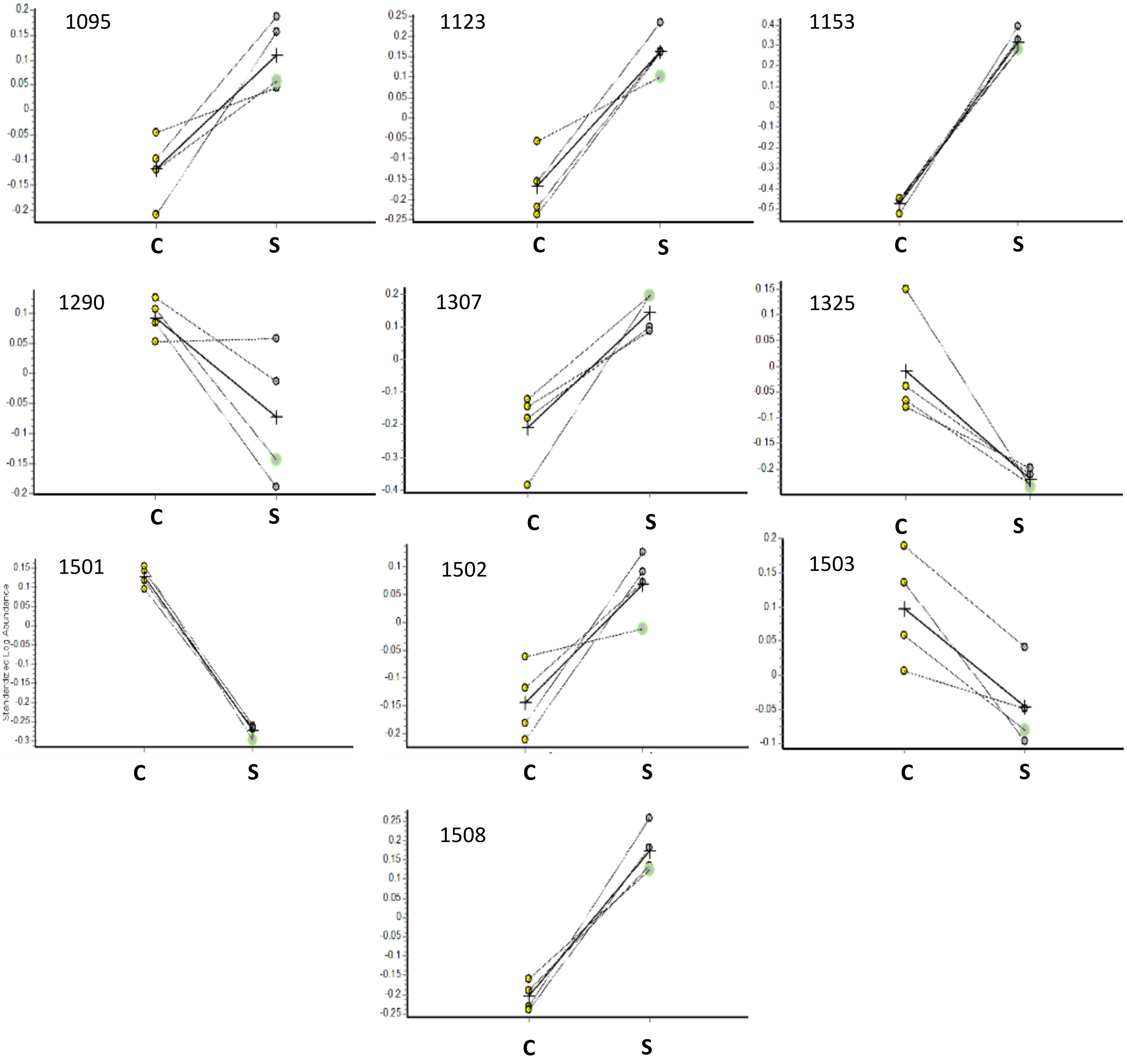

Additional file 1. 2D-DIGE analysis of EBC proteins to identify salt-responsive proteins. (A) A representative multi-plexed gel of EBC proteins from control and salt-treated EBC. Protein was separated by isoelectric focusing on 3 to 10 linear immobilized pH gradient strips for the first dimension and by SDS-PAGE on a 10% linear acrylamide gel for the second dimension and stained with SyproRuby. Differentially expressed proteins are labelled with the respective spot number. (**B**) Graphical representation of the standardized log abundance (i.e., log abundance of Cy3- or Cy5-labeled spot over log abundance of Cy2-labeled standard spot) of the protein spots that showed significant changes in abundance between the untreated and salt-treated samples after analysis with Decyder Software. Individual dashed lines show each of the four biological replicates from untreated (C) and salt-treated (S) replicate samples. The solid line represents the average of the four biological replicates.
