# Supplementary material for: Single-cell-type quantitative proteomic and ionomic analysis of epidermal bladder cells from the halophyte model plant Mesembryanthemum crystallinum to identify salt-responsive proteins
Source: BMC Plant Biol. 2016 May 10;16:110. doi: 10.1186/s12870-016-0797-1 (PMC4862212; doi:10.1186/s12870-016-0797-1)
Supplement: Additional file 2: — Table of additional protein and peptide information from MS analysis of DIGE spots. (PDF 585 kb) [file 12870_2016_797_MOESM2_ESM.pdf]

Additional file 2. Additional information for peptides and proteins identified in the DIGE ana

| Spot # | Protein Name | Accession # | Protein M.W. | Protein Identification Probability | Exclusive unique peptide count | Percentage Sequence Coverage | Peptide Sequence[a] | Best Peptide Identification Probability | Best Mascot Ion Score | Best Mascot Identity Score | Best Mascot Delta Ion Score | Number of Identified +1H spectra | Number of Identified +2H spectra | Number of Identified +3H spectra | Number of Identified +4H spectra |
|---|---|---|---|---|---|---|---|---|---|---|---|---|---|---|---|
| 664 | V-ATPase A subunit | gi\|13235340 | 68900 | 100.00% | 12 | 22.90% | K.EFTMLQSWPVR.T | 97.50% | 45.4 | 43.4 | 23.4 | 0 | 1 | 0 | 0 |
| | | | | | | | K.KIGEGDLLTGGDLYAK.V | 99.70% | 78.1 | 42.1 | 61.9 | 0 | 1 | 0 | 0 |
| | | | | | | | K.LAADTPLLTGQR.V | 99.70% | 76.7 | 39.2 | 53 | 0 | 3 | 0 | 0 |
| | | | | | | | R.LAEMPADSGYPAYLAAR.L | 99.70% | 64.4 | 44.1 | 40.5 | 0 | 1 | 0 | 0 |
| | | | | | | | R.LASFYER.A | 95.80% | 44.5 | 40.7 | 15.5 | 0 | 1 | 0 | 0 |
| | | | | | | | R.LGDLFYR.L | 99.30% | 45.2 | 40.8 | 14.6 | 0 | 2 | 0 | 0 |
| | | | | | | | R.LVSQKFEDPAEGEDTLVAK.F | 99.70% | 57.3 | 44.5 | 21.2 | 0 | 0 | 2 | 0 |
| | | | | | | | K.LYEDLSAAFR.N | 99.70% | 72.1 | 41.9 | 31.1 | 0 | 2 | 0 | 0 |
| | | | | | | | R.SGDVYIPR.G | 99.60% | 49.3 | 41 | 18.3 | 0 | 2 | 0 | 0 |
| | | | | | | | R.TTLVANTSNMPVAAR.E | 99.70% | 104 | 43.4 | 75.6 | 0 | 1 | 0 | 0 |
| | | | | | | | K.TVISQALSK.Y | 98.70% | 47.2 | 39.5 | 18 | 0 | 2 | 0 | 0 |
| | | | | | | | R.VGYDNLIGEIIR.L | 99.70% | 76.3 | 41.3 | 46.8 | 0 | 2 | 0 | 0 |
| 740a | Ferredoxin--nitrite reductase | gi\|255555453 | 65991 | 100.00% | 4 | 7.84% | R.AMKVTEEVQR.L | 98.20% | 39 | 42.2 | 19.2 | 0 | 0 | 1 | 0 |
| | | | | | | | R.GVVLSDVPDILK.G | 99.70% | 63.6 | 38.6 | 47.5 | 0 | 2 | 0 | 0 |
| | | | | | | | R.IGSDSHLGDVYKK.S | 95.70% | 43.3 | 42.6 | 22.4 | 0 | 1 | 0 | 0 |
| | | | | | | | R.VQADDMDELAR.L | 99.70% | 55.8 | 41.9 | 27.1 | 0 | 2 | 0 | 0 |
| 740b | Beta-hexosaminidase 1 | gi\|110742769 | 61231 | 100.00% | 3 | 3.14% | R.GLLIDTSR.H | 98.30% | 47.7 | 42.3 | 0 | 0 | 3 | 0 | 0 |
| | | | | | | | R.GLLIDTSRHYLPIDVIK.Q | 99.40% | 40.8 | 36.6 | 27 | 0 | 0 | 3 | 0 |
| | | | | | | | R.HYLPIDVIK.Q | 97.40% | 31.6 | 37.2 | 11.5 | 0 | 0 | 2 | 0 |
| 760 | V-ATPase B subunit | gi\|26986106 | 54129 | 100.00% | 31 | 73.90% | K.AVVGEEALSSEDLLYLEFLDKFER.K | 95.10% | 41.1 | 43.8 | 30.8 | 0 | 0 | 1 | 0 |
| | | | | | | | K.AVVGEEALSSEDLLYLEFLDKFERK.F | 99.70% | 44.7 | 43 | 33.1 | 0 | 0 | 0 | 2 |
| | | | | | | | K.AVVQVFEGTSGIDNK.Y | 99.70% | 104 | 42.9 | 81.2 | 0 | 1 | 0 | 0 |
| | | | | | | | R.DFEENGSMER.V | 99.00% | 46.4 | 39 | 32.1 | 0 | 2 | 0 | 0 |
| | | | | | | | R.DHSDVSNQLYANYAIGK.D | 99.70% | 90.7 | 45 | 72.3 | 0 | 1 | 0 | 0 |
| | | | | | | | R.DHSDVSNQLYANYAIGKDVQAMK.A | 99.70% | 66.9 | 45.9 | 59 | 0 | 0 | 2 | 0 |
| | | | | | | | R.ELLHRIPAK.T | 98.80% | 40 | 34.8 | 17.1 | 0 | 3 | 0 | 0 |
| | | | | | | | K.FVAQGAYDTR.T | 98.60% | 56 | 44.3 | 32 | 0 | 3 | 0 | 0 |
| | | | | | | | M.GAPNNLEMDEGNLEIGMEYR.T | 99.70% | 53 | 45 | 30.1 | 0 | 0 | 1 | 0 |
| | | | | | | | R.GQKIPLFSAAGLPHNEIAAQICR.Q | 99.70% | 60.5 | 42.1 | 46.6 | 0 | 0 | 3 | 3 |
| | | | | | | | R.GQVLEVDGEKAVVQVFEGTSGIDNK.Y | 99.70% | 69.2 | 43.6 | 0 | 0 | 0 | 1 | 0 |
| | | | | | | | R.GYPGYMYTDLATIYER.A | 99.70% | 101 | 44.6 | 85.9 | 0 | 3 | 0 | 0 |
| | | | | | | | K.HVLVILTDMSSYADALREVSAAR.E | 99.70% | 52.2 | 43.4 | 43 | 0 | 0 | 2 | 1 |
| | | | | | | | R.IALTTAEYLAYECGK.H | 99.70% | 107 | 44.1 | 83.7 | 0 | 3 | 1 | 0 |
| | | | | | | | R.IFNGSGKPIDNGPPILPEAYLDISGSSINPSER.T | 98.90% | 44.6 | 43.6 | 30.2 | 0 | 0 | 1 | 0 |
| | | | | | | | K.IPLFSAAGLPHNEIAAQICR.Q | 99.70% | 69.1 | 42.3 | 59.3 | 0 | 0 | 3 | 0 |
| | | | | | | | R.KFVAQGAYDTR.T | 99.70% | 56.3 | 44.3 | 30.4 | 0 | 1 | 3 | 0 |
| | | | | | | | R.QIYPPINVLPSLSR.L | 99.40% | 50.1 | 38.4 | 34.8 | 0 | 3 | 0 | 0 |
| | | | | | | | R.RDHSDVSNQLYANYAIGKDVQAMK.A | 96.90% | 41.8 | 45.6 | 23.5 | 0 | 0 | 0 | 2 |
| | | | | | | | R.RGQVLEVDGEK.A | 99.70% | 76.3 | 41.6 | 0 | 0 | 2 | 0 | 0 |
| | | | | | | | R.RGQVLEVDGEKAVVQVFEGTSGIDNK.Y | 99.70% | 67.2 | 42.9 | 0 | 0 | 0 | 2 | 2 |
| | | | | | | | K.SAIGEGMTR.R | 97.60% | 48.7 | 42.2 | 27 | 0 | 2 | 0 | 0 |
| | | | | | | | R.TIFQSLDLAWTLLR.I | 99.70% | 103 | 39 | 87.5 | 0 | 3 | 1 | 0 |
| | | | | | | | K.TLDQYYSR.D | 97.80% | 42.6 | 42.1 | 19.7 | 0 | 3 | 0 | 0 |
| | | | | | | | K.TPVSLDMLGR.I | 98.80% | 54.7 | 41.9 | 0 | 0 | 1 | 0 | 0 |
| | | | | | | | R.TVSGVAGPLVILEK.V | 99.70% | 76.5 | 36.8 | 58.8 | 0 | 3 | 0 | 0 |
| | | | | | | | R.TYPEEMIQTGISTIDVMNSIAR.G | 99.70% | 70.9 | 45.9 | 55.9 | 0 | 3 | 3 | 0 |
| | | | | | | | R.VTLFLNLANDPTIER.I | 99.70% | 95.3 | 41 | 13.6 | 0 | 3 | 1 | 0 |
| | | | | | | | K.YQEIVNIR.L | 99.70% | 60.1 | 41.2 | 0 | 0 | 3 | 0 | 0 |
| | | | | | | | K.YTTVQFTGEVLK.T | 99.70% | 83 | 42.1 | 62.2 | 0 | 1 | 0 | 0 |

| | | | | | | | | | | | | | | | |
|---|---|---|---|---|---|---|---|---|---|---|---|---|---|---|---|
| | | | | | | | K.YTTVQFTGEVLKTPVSLDMLGR.I | 99.70% | 68.5 | 42.7 | 64.7 | 0 | 0 | 3 | 0 |
| 765 | NADP-dependent malic enzyme | gi\|585453 | 64360 | 100.00% | 46 | 70.60% | K.APVEQMR.K | 99.20% | 46.5 | 38.9 | 25 | 0 | 3 | 0 | 0 |
| | | | | | | | R.ATGEEYAEFVQEFMSAVK.Q | 99.70% | 102 | 45 | 91 | 0 | 4 | 3 | 0 |
| | | | | | | | R.ATGEEYAEFVQEFMSAVKQNYGEK.I | 99.70% | 86.8 | 46.5 | 74.6 | 0 | 0 | 2 | 0 |
| | | | | | | | K.AYELGLASR.L | 99.70% | 63.7 | 41 | 29.9 | 0 | 1 | 0 | 0 |
| | | | | | | | K.AYELGLASRLPQPADLVK.F | 99.70% | 47.4 | 39.5 | 38.7 | 0 | 1 | 3 | 0 |
| | | | | | | | R.DAHFLR.G | 97.30% | 38.2 | 39 | 2.55 | 0 | 2 | 0 | 0 |
| | | | | | | | K.ERDAHFLR.G | 97.10% | 35.3 | 43.3 | 0 | 0 | 1 | 0 | 0 |
| | | | | | | | K.FAESCMYNPTYR.S | 99.70% | 79.1 | 42.2 | 60.7 | 0 | 6 | 2 | 0 |
| | | | | | | | K.FLFLGAGEAGTGIAELIALEMSKK.T | 97.50% | 38.8 | 40 | 27.3 | 0 | 0 | 1 | 0 |
| | | | | | | | K.FLTTLR.Q | 99.30% | 35.9 | 35.5 | 0 | 0 | 3 | 0 | 0 |
| | | | | | | | K.GLAFTEK.E | 98.20% | 35.8 | 41 | 17.5 | 0 | 2 | 0 | 0 |
| | | | | | | | K.GLAFTEKER.D | 99.70% | 61.8 | 41.7 | 33.3 | 0 | 2 | 0 | 0 |
| | | | | | | | K.GLIYPPFKDIR.K | 96.10% | 35.8 | 39.1 | 0 | 0 | 1 | 0 | 0 |
| | | | | | | | K.GLIYPPFKDIRK.I | 94.70% | 22.6 | 37.1 | 0 | 0 | 0 | 1 | 0 |
| | | | | | | | R.GLLPPVVLSQELQEK.K | 99.70% | 95.5 | 36.6 | 81.2 | 0 | 5 | 2 | 0 |
| | | | | | | | R.GLLPPVVLSQELQEKK.F | 99.70% | 72 | 34.1 | 44.8 | 0 | 3 | 0 | 0 |
| | | | | | | | K.GLVVSSRK.E | 96.70% | 31.1 | 39.9 | 11.8 | 0 | 1 | 0 | 0 |
| | | | | | | | R.ILGLGDLGCQGMGIPVGK.L | 99.70% | 66.5 | 42.8 | 28.6 | 0 | 3 | 3 | 0 |
| | | | | | | | K.ILVQFEDFANHNAFELLEK.Y | 99.70% | 83.5 | 44 | 4.96 | 0 | 2 | 3 | 0 |
| | | | | | | | K.ILVQFEDFANHNAFELLEKYR.T | 99.70% | 71.4 | 43.4 | 11.1 | 0 | 0 | 2 | 0 |
| | | | | | | | K.IQVIVVTDGER.I | 99.50% | 43 | 40.3 | 32.9 | 0 | 3 | 0 | 0 |
| | | | | | | | K.ISAHIAAGVAAK.A | 99.70% | 42.9 | 37.7 | 18.6 | 0 | 0 | 3 | 0 |
| | | | | | | | K.IWLVDSK.G | 98.60% | 37.7 | 41.3 | 9.39 | 0 | 2 | 0 | 0 |
| | | | | | | | R.KETLQQFK.L | 95.70% | 43.9 | 41.5 | 21.4 | 0 | 1 | 0 | 0 |
| | | | | | | | K.KIQVIVVTDGER.I | 99.70% | 71.7 | 38.6 | 50.4 | 0 | 2 | 0 | 0 |
| | | | | | | | R.KISAHIAAGVAAK.A | 99.70% | 56.3 | 34.1 | 10.9 | 0 | 3 | 3 | 0 |
| | | | | | | | K.KIWLVDSK.G | 98.30% | 35.1 | 39.9 | 17.6 | 0 | 2 | 0 | 0 |
| | | | | | | | R.KKIWLVDSK.G | 97.40% | 29.8 | 37.1 | 10.4 | 0 | 1 | 0 | 0 |
| | | | | | | | K.LLDDEFYIGLK.Q | 99.70% | 87.7 | 45.4 | 57.5 | 0 | 3 | 0 | 0 |
| | | | | | | | K.LLVDHVEELLPLVYTPTVGEGCQK.Y | 99.70% | 78.3 | 41.8 | 66.2 | 0 | 0 | 2 | 2 |
| | | | | | | | R.LPQPADLVK.F | 99.70% | 61.8 | 36.9 | 37.8 | 0 | 5 | 0 | 0 |
| | | | | | | | K.LPWAHEHEPITTLIDAVQAIKPTVLIGTSGK.G | 99.70% | 66.2 | 35.9 | 50.8 | 0 | 0 | 0 | 2 |
| | | | | | | | K.LSLYSALGGVCPSACLPITLDVGTNNQK.L | 99.70% | 52 | 44.2 | 37.2 | 0 | 0 | 2 | 0 |
| | | | | | | | R.NEKLFYK.L | 97.30% | 37.6 | 38.5 | 8.72 | 0 | 1 | 0 | 0 |
| | | | | | | | R.QYQVPLQK.Y | 99.40% | 51.4 | 41.8 | 22.8 | 0 | 3 | 0 | 0 |
| | | | | | | | K.RATGEEYAEFVQEFMSAVK.Q | 99.70% | 71.5 | 45.3 | 55.5 | 0 | 0 | 3 | 0 |
| | | | | | | | R.RPQGLFISLK.D | 99.60% | 41.7 | 37.5 | 7.84 | 0 | 3 | 0 | 0 |
| | | | | | | | R.RPQGLFISLKDK.G | 99.70% | 56.9 | 37.3 | 39 | 0 | 1 | 3 | 0 |
| | | | | | | | R.RPQGLFISLKDKGR.I | 99.70% | 38.6 | 35.8 | 30.4 | 0 | 0 | 2 | 2 |
| | | | | | | | K.TKAPVEQMR.K | 98.50% | 37.3 | 41.3 | 20.4 | 0 | 1 | 0 | 0 |
| | | | | | | | R.TTHLVFNDDIQGTASVVLAGLIASLK.L | 99.70% | 62.4 | 37.5 | 13.8 | 0 | 0 | 1 | 0 |
| | | | | | | | R.VHDDMLLAASEALASQVTGEHFIK.G | 99.70% | 87.1 | 44.6 | 78.2 | 0 | 3 | 4 | 5 |
| | | | | | | | K.YGSIFR.R | 99.30% | 38.5 | 37.4 | 0 | 0 | 3 | 0 | 0 |
| | | | | | | | K.YMAMMDLQER.N | 99.40% | 43.2 | 41.6 | 1.11 | 0 | 4 | 0 | 0 |
| | | | | | | | K.YMAMMDLQERNEK.L | 95.70% | 29.7 | 43.4 | 18 | 0 | 1 | 1 | 0 |
| | | | | | | | K.YMAMMDLQERNEKLFYK.L | 95.40% | 31.6 | 45.2 | 22.4 | 0 | 0 | 0 | 1 |
| 767 | NADP-dependent malic enzyme | gi\|585453 | 64360 | 100.00% | 49 | 76.90% | K.APVEQMR.K | 99.60% | 44.5 | 38.9 | 22.9 | 0 | 3 | 0 | 0 |
| | | | | | | | R.ATGEEYAEFVQEFMSAVK.Q | 99.70% | 77.4 | 45 | 60.5 | 0 | 3 | 4 | 0 |
| | | | | | | | R.ATGEEYAEFVQEFMSAVKQNYGEK.I | 99.70% | 73.8 | 46.5 | 63.1 | 0 | 0 | 2 | 0 |
| | | | | | | | K.AYELGLASR.L | 99.70% | 62.1 | 41.1 | 29 | 0 | 1 | 0 | 0 |
| | | | | | | | K.AYELGLASRLPQPADLVK.F | 99.70% | 52.5 | 39.6 | 41.9 | 0 | 0 | 3 | 0 |
| | | | | | | | K.ERDAHFLR.G | 97.50% | 36.5 | 43.3 | 7.39 | 0 | 2 | 0 | 0 |
| | | | | | | | K.ETLQQFK.L | 99.30% | 42.4 | 41.2 | 14.6 | 0 | 3 | 0 | 0 |
| | | | | | | | K.ETLQQFKLPWAHEHEPITTLIDAVQAIKPTVLIGTSGK.L | 99.30% | 35.8 | 37.5 | 20.4 | 0 | 0 | 0 | 1 |

| | | | | | | | | | | | | | | | |
|---|---|---|---|---|---|---|---|---|---|---|---|---|---|---|---|
| | | | | | | | K.FAESCMYNPTYR.S | 99.70% | 83.4 | 42.1 | 61.4 | 0 | 6 | 1 | 0 |
| | | | | | | | K.FLFLGAGEAGTGIAELIALEMSK.K | 99.70% | 87.6 | 42.3 | 74.1 | 0 | 0 | 3 | 0 |
| | | | | | | | K.FLFLGAGEAGTGIAELIALEMSKK.T | 99.70% | 48.5 | 40 | 29.1 | 0 | 0 | 3 | 0 |
| | | | | | | | K.FLTTLR.Q | 97.80% | 36.7 | 36 | 0 | 0 | 3 | 0 | 0 |
| | | | | | | | K.GLAFTEKER.D | 99.50% | 53.8 | 41.6 | 26.1 | 0 | 1 | 0 | 0 |
| | | | | | | | K.GLIYPPFKDIR.K | 98.10% | 40.8 | 39.3 | 0 | 0 | 1 | 0 | 0 |
| | | | | | | | R.GLLPPVVLSQELQEK.K | 99.70% | 81.7 | 36.7 | 67.2 | 0 | 4 | 3 | 0 |
| | | | | | | | R.GLLPPVVLSQELQEKK.F | 99.70% | 88.4 | 34.1 | 54.5 | 0 | 3 | 0 | 0 |
| | | | | | | | K.GLVVSSRK.E | 98.80% | 38 | 39.9 | 20.4 | 0 | 3 | 0 | 0 |
| | | | | | | | R.ILGLGDLGCQGMGIPVGK.L | 99.70% | 67.2 | 42.9 | 37.3 | 0 | 3 | 3 | 0 |
| | | | | | | | K.ILVQFEDFANHNAFELLEK.Y | 99.70% | 68.5 | 44.1 | 2.78 | 0 | 1 | 3 | 0 |
| | | | | | | | K.ILVQFEDFANHNAFELLEKYR.T | 99.70% | 56.1 | 43.4 | 9.08 | 0 | 0 | 3 | 1 |
| | | | | | | | K.IQVIVVTDGER.I | 98.10% | 41.9 | 40.4 | 31.8 | 0 | 2 | 0 | 0 |
| | | | | | | | K.IQVIVVTDGERILGLGDLGCQGMGIPVGK.L | 99.50% | 34.5 | 39.8 | 21.1 | 0 | 0 | 1 | 0 |
| | | | | | | | K.ISAHIAAGVAAK.A | 99.70% | 43.5 | 37.7 | 19.3 | 0 | 0 | 3 | 0 |
| | | | | | | | K.IWLVDSK.G | 97.00% | 39.8 | 41.3 | 6.47 | 0 | 2 | 0 | 0 |
| | | | | | | | K.KIQVIVVTDGER.I | 99.60% | 58.8 | 38.8 | 34.6 | 0 | 1 | 1 | 0 |
| | | | | | | | R.KISAHIAAGVAAK.A | 99.70% | 83.6 | 34.1 | 37.8 | 0 | 3 | 3 | 0 |
| | | | | | | | K.KIWLVDSK.G | 99.50% | 51.5 | 40.2 | 27.2 | 0 | 2 | 0 | 0 |
| | | | | | | | R.KKIWLVDSK.G | 98.60% | 34 | 37.1 | 11.2 | 0 | 3 | 0 | 0 |
| | | | | | | | K.LLDDEFYIGLK.Q | 99.70% | 87.6 | 45.4 | 57.6 | 0 | 3 | 0 | 0 |
| | | | | | | | K.LLGGTLADHKFLFLGAGEAGTGIAELIALEMSK.K | 99.70% | 41.5 | 39 | 5.2 | 0 | 0 | 0 | 1 |
| | | | | | | | K.LLGGTLADHKFLFLGAGEAGTGIAELIALEMSKK.T | 99.70% | 57.5 | 38.7 | 47 | 0 | 0 | 0 | 3 |
| | | | | | | | K.LLVDHVEELLPLVYTPTVGEGCQK.Y | 99.70% | 58.8 | 41.9 | 46.1 | 0 | 0 | 0 | 1 |
| | | | | | | | R.LPQPADLVK.F | 99.70% | 65.3 | 38.2 | 41 | 0 | 5 | 0 | 0 |
| | | | | | | | K.LPWAHEHEPITTLIDAVQAIKPTVLIGTSGK.G | 99.70% | 66.2 | 35.8 | 59 | 0 | 0 | 0 | 2 |
| | | | | | | | K.LSLYSALGGVCPSACLPITLDVGTNNQK.L | 99.70% | 51.9 | 44.3 | 46.6 | 0 | 0 | 2 | 0 |
| | | | | | | | R.NEKLFYK.L | 99.00% | 37.9 | 38.5 | 6.86 | 0 | 3 | 0 | 0 |
| | | | | | | | R.QYQVPLQK.Y | 98.30% | 37.3 | 41.8 | 12.4 | 0 | 3 | 0 | 0 |
| | | | | | | | K.RATGEEYAEFVQEFMSAVK.Q | 99.70% | 68.4 | 45.2 | 48.3 | 0 | 0 | 3 | 0 |
| | | | | | | | R.RPQGLFISLK.D | 99.70% | 39.7 | 37.6 | 29.5 | 0 | 3 | 1 | 0 |
| | | | | | | | R.RPQGLFISLKDK.G | 99.70% | 55.6 | 37.7 | 38.6 | 0 | 1 | 3 | 0 |
| | | | | | | | R.RPQGLFISLKDKGR.I | 99.70% | 65.9 | 36.4 | 43.3 | 0 | 0 | 3 | 3 |
| | | | | | | | R.TFVPGQANNAYIFPGFGLGLIMCGAIR.V | 99.70% | 71.5 | 44.1 | 57 | 0 | 0 | 6 | 0 |
| | | | | | | | K.TKAPVEQMR.K | 99.00% | 40.5 | 41.3 | 22 | 0 | 1 | 0 | 0 |
| | | | | | | | R.TTHLVFNDDIQGTASVVLAGLIASLK.L | 99.70% | 111 | 37.8 | 0 | 0 | 1 | 3 | 2 |
| | | | | | | | R.VHDDMLLAASEALASQVTGEHFIK.G | 99.70% | 96 | 44.5 | 82.7 | 0 | 2 | 3 | 3 |
| | | | | | | | K.YGSIFR.R | 98.60% | 34.2 | 37.5 | 0 | 0 | 3 | 0 | 0 |
| | | | | | | | K.YMAMMDLQER.N | 99.60% | 46 | 41.2 | 30.2 | 0 | 4 | 0 | 0 |
| | | | | | | | K.YMAMMDLQERNEK.L | 99.20% | 36.3 | 43.5 | 24 | 0 | 1 | 1 | 0 |
| | | | | | | | K.YRTTHLVFNDDIQGTASVVLAGLIASLK.L | 99.70% | 42.2 | 40.3 | 7.13 | 0 | 0 | 0 | 1 |
| 774 | NADP-dependent malic enzyme | gi\|585453 | 64360 | 100.00% | 48 | 75.90% | K.APVEQMR.K | 99.30% | 43.8 | 38.9 | 21.6 | 0 | 3 | 0 | 0 |
| | | | | | | | R.ATGEEYAEFVQEFMSAVK.Q | 99.70% | 107 | 44.9 | 97.5 | 0 | 3 | 3 | 0 |
| | | | | | | | R.ATGEEYAEFVQEFMSAVKQNYGEK.I | 99.70% | 71.9 | 46.5 | 61.8 | 0 | 0 | 3 | 0 |
| | | | | | | | K.AYELGLASR.L | 99.70% | 70.4 | 40.5 | 36.2 | 0 | 2 | 0 | 0 |
| | | | | | | | K.AYELGLASRLPQPADLVK.F | 99.70% | 44.7 | 40.2 | 31.9 | 0 | 1 | 3 | 0 |
| | | | | | | | R.DAHFLR.G | 95.40% | 31 | 39.3 | 0.07 | 0 | 1 | 0 | 0 |
| | | | | | | | K.ERDAHFLR.G | 96.20% | 35.9 | 43.3 | 7.07 | 0 | 2 | 0 | 0 |
| | | | | | | | K.ETLQQFK.L | 99.30% | 42.8 | 41.2 | 14.6 | 0 | 1 | 0 | 0 |
| | | | | | | | K.ETLQQFKLPWAHEHEPITTLIDAVQAIKPTVLIGTSGK.G | 99.00% | 34.8 | 37.7 | 24.6 | 0 | 0 | 0 | 0 |
| | | | | | | | K.FAESCMYNPTYR.S | 99.70% | 72.1 | 42 | 54.1 | 0 | 6 | 1 | 0 |
| | | | | | | | K.FLFLGAGEAGTGIAELIALEMSKK.T | 98.70% | 36.2 | 41.2 | 17.2 | 0 | 0 | 1 | 0 |
| | | | | | | | K.GLAFTEKER.D | 99.70% | 57.5 | 41.6 | 37 | 0 | 1 | 0 | 0 |
| | | | | | | | K.GLAFTEKERDAHFLR.G | 98.90% | 35.3 | 42.6 | 29.4 | 0 | 0 | 2 | 0 |
| | | | | | | | K.GLIYPPFKDIR.K | 96.60% | 37.1 | 39.4 | 0 | 0 | 2 | 0 | 0 |
| | | | | | | | R.GLLPPVVLSQELQEK.K | 99.70% | 88.8 | 37.1 | 60.2 | 0 | 5 | 3 | 0 |

| | | | | | | | | | | | | | | | |
|---|---|---|---|---|---|---|---|---|---|---|---|---|---|---|---|
| | | | | | | | R.GLLPPVVLSQELQEKK.F | 99.70% | 81.6 | 34.6 | 52.1 | 0 | 3 | 0 | 0 |
| | | | | | | | K.GLVVSSRK.E | 95.80% | 39 | 39.9 | 21.3 | 0 | 1 | 0 | 0 |
| | | | | | | | R.ILGLGDLGCQGMGIPVGK.L | 99.70% | 68.8 | 43 | 27 | 0 | 3 | 3 | 0 |
| | | | | | | | K.ILVQFEDFANHNAFELLEK.Y | 99.70% | 64.3 | 43.8 | 2.15 | 0 | 2 | 3 | 0 |
| | | | | | | | K.ILVQFEDFANHNAFELLEKYR.T | 99.70% | 62.6 | 43.7 | 10.9 | 0 | 0 | 3 | 0 |
| | | | | | | | K.IQVIVVTDGER.I | 99.70% | 62.8 | 40.9 | 43.8 | 0 | 1 | 0 | 0 |
| | | | | | | | K.ISAHIAAGVAAK.A | 99.70% | 74.2 | 37.7 | 32.3 | 0 | 3 | 3 | 0 |
| | | | | | | | K.IWLVDSK.G | 97.50% | 41.7 | 42.1 | 0 | 0 | 1 | 0 | 0 |
| | | | | | | | R.KETLQQFK.L | 98.00% | 40.4 | 41.5 | 15.2 | 0 | 1 | 0 | 0 |
| | | | | | | | K.KIQVIVVTDGER.I | 99.70% | 59.2 | 39.4 | 31.5 | 0 | 3 | 1 | 0 |
| | | | | | | | R.KISAHIAAGVAAK.A | 99.70% | 85.3 | 34.1 | 36.3 | 0 | 3 | 3 | 0 |
| | | | | | | | K.KIWLVDSK.G | 98.30% | 42.8 | 40.1 | 22.6 | 0 | 2 | 0 | 0 |
| | | | | | | | R.KKIWLVDSK.G | 99.00% | 37.2 | 37.1 | 14.2 | 0 | 2 | 0 | 0 |
| | | | | | | | K.LLDDEFYIGLK.Q | 99.70% | 87.7 | 45.5 | 57.6 | 0 | 3 | 0 | 0 |
| | | | | | | | K.LLGGTLADHKFLFLGAGEAGTGIAELIALEMSK.K | 99.70% | 46 | 39.4 | 15.6 | 0 | 0 | 0 | 1 |
| | | | | | | | K.LLVDHVEELLPLVYTPTVGEGCQK.Y | 98.00% | 33 | 42.2 | 21.8 | 0 | 0 | 0 | 1 |
| | | | | | | | R.LPQPADLVK.F | 99.70% | 64 | 37.3 | 41.2 | 0 | 6 | 0 | 0 |
| | | | | | | | K.LPWAHEHEPITTLIDAVQAIKPTVLIGTSGK.G | 99.70% | 69.3 | 37.1 | 61.8 | 0 | 0 | 0 | 2 |
| | | | | | | | K.LSLYSALGGVCPSACLPITLDVGTNNQK.L | 99.70% | 54.2 | 44.5 | 45.2 | 0 | 0 | 3 | 0 |
| | | | | | | | R.NEKLFYK.L | 97.70% | 36.5 | 38.5 | 5.39 | 0 | 1 | 0 | 0 |
| | | | | | | | R.QYQVPLQK.Y | 98.90% | 41 | 41.7 | 5.32 | 0 | 3 | 0 | 0 |
| | | | | | | | K.RATGEEYAEFVQEFMSAVK.Q | 99.70% | 63.8 | 45.2 | 45.4 | 0 | 0 | 3 | 0 |
| | | | | | | | K.RATGEEYAEFVQEFMSAVKQNYGEK.I | 97.30% | 35.7 | 46.2 | 17.5 | 0 | 0 | 0 | 1 |
| | | | | | | | R.RPQGLFISLK.D | 98.70% | 41.3 | 37.5 | 12.3 | 0 | 3 | 0 | 0 |
| | | | | | | | R.RPQGLFISLKDK.G | 99.70% | 43.5 | 38.5 | 25.9 | 0 | 2 | 3 | 0 |
| | | | | | | | R.RPQGLFISLKDKGR.I | 99.50% | 54.2 | 36.8 | 35.4 | 0 | 1 | 1 | 3 |
| | | | | | | | R.TFVPGQANNAYIFPGFGLGLIMCGAIR.V | 99.70% | 64.8 | 44.3 | 52.5 | 0 | 0 | 3 | 0 |
| | | | | | | | K.TKAPVEQMR.K | 96.10% | 37.1 | 42 | 26.2 | 0 | 1 | 0 | 0 |
| | | | | | | | R.TTHLVFNDDIQGTASVVLAGLIASLK.L | 99.70% | 46 | 38.6 | 9.17 | 0 | 0 | 3 | 0 |
| | | | | | | | R.VHDDMLLAASEALASQVTGEHFIK.G | 99.70% | 90.5 | 44.5 | 82.9 | 0 | 2 | 5 | 4 |
| | | | | | | | K.YGSIFR.R | 98.00% | 38.5 | 37.7 | 0 | 0 | 3 | 0 | 0 |
| | | | | | | | K.YMAMMDLQER.N | 99.30% | 43.4 | 41.2 | 31.1 | 0 | 5 | 0 | 0 |
| | | | | | | | K.YMAMMDLQERNEK.L | 99.20% | 44.7 | 43.5 | 33.5 | 0 | 2 | 0 | 0 |
| 775 | NADP-dependent malic enzyme | gi\|585453 | 64360 | 100.00% | 54 | 76.90% | K.APVEQMR.K | 99.60% | 47.1 | 38.9 | 25.6 | 0 | 3 | 0 | 0 |
| | | | | | | | R.ATGEEYAEFVQEFMSAVK.Q | 99.70% | 108 | 45 | 98 | 0 | 3 | 5 | 0 |
| | | | | | | | R.ATGEEYAEFVQEFMSAVKQNYGEK.I | 99.70% | 82.7 | 46.5 | 75.8 | 0 | 0 | 4 | 0 |
| | | | | | | | K.AYELGLASR.L | 99.70% | 61.7 | 41.1 | 30.9 | 0 | 3 | 0 | 0 |
| | | | | | | | K.AYELGLASRLPQPADLVK.F | 99.70% | 50 | 39.4 | 40.6 | 0 | 0 | 3 | 0 |
| | | | | | | | R.DAHFLR.G | 97.00% | 33.6 | 39.3 | 0.07 | 0 | 1 | 0 | 0 |
| | | | | | | | K.ERDAHFLR.G | 96.40% | 36.4 | 43.4 | 14 | 0 | 1 | 0 | 0 |
| | | | | | | | K.ETLQQFK.L | 99.10% | 43.5 | 41.2 | 9.38 | 0 | 2 | 0 | 0 |
| | | | | | | | K.ETLQQFKLPWAHEHEPITTLIDAVQAIKPTVLIGTSGK.G | 99.70% | 40.1 | 37.3 | 33.6 | 0 | 0 | 0 | 0 |
| | | | | | | | K.FAESCMYNPTYR.S | 99.70% | 81 | 42.2 | 63 | 0 | 6 | 1 | 0 |
| | | | | | | | K.FLFLGAGEAGTGIAELIALEMSK.K | 99.00% | 40.4 | 42.1 | 27.9 | 0 | 0 | 1 | 0 |
| | | | | | | | K.FLFLGAGEAGTGIAELIALEMSKK.T | 99.60% | 47.3 | 40 | 24.9 | 0 | 0 | 2 | 0 |
| | | | | | | | K.FLTTLR.Q | 98.80% | 36.2 | 36 | 0 | 0 | 1 | 0 | 0 |
| | | | | | | | K.FLTTLRQYQVPLQK.Y | 96.20% | 25.6 | 37.6 | 17.2 | 0 | 0 | 1 | 0 |
| | | | | | | | K.GLAFTEKER.D | 99.70% | 61.3 | 41.7 | 41 | 0 | 1 | 0 | 0 |
| | | | | | | | K.GLAFTEKERDAHFLR.G | 98.20% | 33.2 | 42.5 | 24.1 | 0 | 0 | 1 | 0 |
| | | | | | | | K.GLIYPPFK.D | 98.10% | 36.5 | 39.1 | 0 | 0 | 1 | 0 | 0 |
| | | | | | | | K.GLIYPPFKDIR.K | 99.20% | 42.6 | 39.3 | 0 | 0 | 3 | 0 | 0 |
| | | | | | | | R.GLLPPVVLSQELQEK.K | 99.70% | 62.1 | 36.7 | 46.7 | 0 | 5 | 3 | 0 |
| | | | | | | | R.GLLPPVVLSQELQEKK.F | 99.70% | 77.2 | 33.7 | 67.1 | 0 | 3 | 0 | 0 |
| | | | | | | | K.GLVVSSRK.E | 96.60% | 38.2 | 39.9 | 16.6 | 0 | 1 | 0 | 0 |
| | | | | | | | R.ILGLGDLGCQGMGIPVGK.L | 99.70% | 76.5 | 42.8 | 54.3 | 0 | 3 | 3 | 0 |
| | | | | | | | K.ILVQFEDFANHNAFELLEK.Y | 99.70% | 82.8 | 44 | 2.87 | 0 | 1 | 1 | 0 |

| | | | | | | | | | | | | | | | |
|---|---|---|---|---|---|---|---|---|---|---|---|---|---|---|---|
| | | | | | | | K.ILVQFEDFANHNAFELLEKYR.T | 99.70% | 61.7 | 43.3 | 14.9 | 0 | 0 | 3 | 0 |
| | | | | | | | K.IQVIVVTDGER.I | 99.70% | 75.2 | 40.4 | 49.6 | 0 | 2 | 0 | 0 |
| | | | | | | | K.IQVIVVTDGERILGLGDLGCQGMGIPVGK.L | 99.70% | 52.9 | 39.8 | 34.1 | 0 | 0 | 1 | 0 |
| | | | | | | | K.ISAHIAAGVAAK.A | 99.70% | 45.5 | 37.7 | 17.8 | 0 | 0 | 3 | 0 |
| | | | | | | | K.IWLVDSK.G | 96.60% | 39.7 | 41.3 | 11.9 | 0 | 2 | 0 | 0 |
| | | | | | | | R.KETLQQFK.L | 98.80% | 42.1 | 41.5 | 24.5 | 0 | 1 | 0 | 0 |
| | | | | | | | R.KETLQQFKLPWAHEHEPITTLIDAVQAIKPTVLIGTSGK.G | 98.40% | 30.8 | 38.5 | 21 | 0 | 0 | 0 | 0 |
| | | | | | | | K.KIQVIVVTDGER.I | 99.70% | 71.9 | 38.6 | 50.3 | 0 | 2 | 2 | 0 |
| | | | | | | | R.KISAHIAAGVAAK.A | 99.70% | 85.2 | 34.1 | 33.9 | 0 | 3 | 3 | 0 |
| | | | | | | | K.KIWLVDSK.G | 99.70% | 50.2 | 39.9 | 28.7 | 0 | 3 | 0 | 0 |
| | | | | | | | R.KKIWLVDSK.G | 97.00% | 36.5 | 37.1 | 10.6 | 0 | 1 | 0 | 0 |
| | | | | | | | K.LLDDEFYIGLK.Q | 99.70% | 87.7 | 45.4 | 57.6 | 0 | 3 | 0 | 0 |
| | | | | | | | K.LLGGTLADHKFLFLGAGEAGTGIAELIALEMSK,K | 99.70% | 50.4 | 38.8 | 4.87 | 0 | 0 | 0 | 1 |
| | | | | | | | K.LLGGTLADHKFLFLGAGEAGTGIAELIALEMSKK.T | 99.70% | 66.8 | 38.5 | 60 | 0 | 0 | 0 | 4 |
| | | | | | | | K.LLVDHVEELLPLVYTPTVGEGCQK.Y | 99.70% | 50.2 | 41.8 | 39 | 0 | 0 | 0 | 1 |
| | | | | | | | R.LPQPADLVK.F | 99.70% | 57.3 | 38.2 | 35 | 0 | 4 | 0 | 0 |
| | | | | | | | K.LPWAHEHEPITTLIDAVQAIKPTVLIGTSGK.G | 99.70% | 78.3 | 35.8 | 75.1 | 0 | 0 | 0 | 2 |
| | | | | | | | K.LSLYSALGGVCPSACLPITLDVGTNNQK.L | 99.70% | 66.6 | 44.1 | 59.2 | 0 | 0 | 3 | 0 |
| | | | | | | | R.NEKLFYK.L | 98.30% | 36.3 | 38.5 | 5.51 | 0 | 1 | 0 | 0 |
| | | | | | | | R.QYQVPLQK.Y | 98.90% | 42.7 | 41.8 | 19.2 | 0 | 3 | 0 | 0 |
| | | | | | | | K.RATGEEYAEFVQEFMSAVK.Q | 99.70% | 68.7 | 45.3 | 48.6 | 0 | 0 | 3 | 0 |
| | | | | | | | R.RPQGLFISLK.D | 98.50% | 42 | 37.5 | 12.7 | 0 | 3 | 0 | 0 |
| | | | | | | | R.RPQGLFISLKDK.G | 99.70% | 47.7 | 37.7 | 31 | 0 | 3 | 3 | 0 |
| | | | | | | | R.RPQGLFISLKDKGR.I | 99.70% | 63.8 | 35.9 | 47.8 | 0 | 0 | 3 | 3 |
| | | | | | | | R.TFVPGQANNAYIFPGFGLGLIMCGAIR.V | 99.70% | 67.7 | 43.8 | 58.8 | 0 | 0 | 2 | 0 |
| | | | | | | | K.TKAPVEQMR.K | 97.00% | 35.3 | 41.3 | 17.4 | 0 | 1 | 0 | 0 |
| | | | | | | | R.TTHLVFNDDIQGTASVVLAGLIASLK.L | 99.70% | 57.5 | 37.7 | 13.5 | 0 | 1 | 3 | 2 |
| | | | | | | | R.VHDDMLLAASEALASQVTGEHFIK.G | 99.70% | 76.5 | 44.5 | 62.2 | 0 | 2 | 3 | 5 |
| | | | | | | | K.YGSIFR.R | 98.20% | 40.5 | 37.4 | 0 | 0 | 3 | 0 | 0 |
| | | | | | | | K.YMAMMDLQER.N | 99.20% | 45.5 | 41.6 | 10.2 | 0 | 7 | 0 | 0 |
| | | | | | | | K.YMAMMDLQERNEK.L | 96.80% | 37.1 | 43.4 | 25.3 | 0 | 1 | 0 | 0 |
| 1062 | Alpha-1,4-glucan-protein synthase | gi\|356495127 | 41713 | 100.00% | 16 | 41.80% | K.ASCISFK.D | 99.10% | 46.8 | 41.7 | 15.2 | 0 | 3 | 0 | 0 |
| | | | | | | | K.ASCISFKDSACR.C | 99.50% | 52.8 | 43 | 35 | 0 | 3 | 1 | 0 |
| | | | | | | | K.ASNPFVNLK.K | 99.20% | 59.5 | 41.5 | 35.7 | 0 | 3 | 0 | 0 |
| | | | | | | | R.CFGYMVSK.K | 97.60% | 37.9 | 40.9 | 18.7 | 0 | 2 | 0 | 0 |
| | | | | | | | R.CFGYMVSKK.K | 95.70% | 34.2 | 41.7 | 22.8 | 0 | 1 | 0 | 0 |
| | | | | | | | K.ECTSVQK.C | 99.00% | 43.5 | 39.7 | 14.1 | 0 | 12 | 0 | 0 |
| | | | | | | | R.EGAPTAVSHGLWLNIPDYDAPTQLVKPLER.N | 99.70% | 116 | 41.8 | 33.2 | 0 | 0 | 3 | 3 |
| | | | | | | | K.GTLFPMCGMNLAFDR.Q | 99.70% | 87 | 44 | 25.6 | 0 | 6 | 3 | 0 |
| | | | | | | | R.GYPFSLREGAPTAVSHGLWLNIPDYDAPTQLVKPLER.N | 99.70% | 58.5 | 40.9 | 4.09 | 0 | 0 | 0 | 2 |
| | | | | | | | K.KKYIYTIDDDCFVAK.D | 97.60% | 35.3 | 44.1 | 21.3 | 0 | 0 | 2 | 0 |
| | | | | | | | K.KYIYTIDDDCFVAK.D | 99.60% | 73.4 | 44 | 56.3 | 0 | 1 | 0 | 0 |
| | | | | | | | K.NLLCPSTPFFFNTLYDPYR.E | 99.70% | 85 | 45.4 | 69.9 | 0 | 3 | 0 | 0 |
| | | | | | | | K.VICDHLGLGVK.T | 99.70% | 76.6 | 41.8 | 56.6 | 0 | 3 | 3 | 0 |
| | | | | | | | R.YDDMWAGWCVK.V | 99.70% | 60.9 | 41.3 | 39.8 | 0 | 6 | 0 | 0 |
| | | | | | | | K.YIYTIDDDCFVAK.D | 99.70% | 78.3 | 44.2 | 58 | 0 | 3 | 0 | 0 |
| | | | | | | | R.YVDAVLTIPK.G | 99.70% | 65.5 | 39.5 | 44.2 | 0 | 3 | 0 | 0 |
| 1065 | Beta-D-galactosidase | gi\|61162194 | 100047 | 100.00% | 3 | 3.65% | K.DLHAAIK.L | 96.40% | 31 | 38.2 | 0 | 0 | 1 | 0 | 0 |
| | | | | | | | K.LGPKQEAHVYR.M | 99.70% | 44.6 | 41.5 | 34.4 | 0 | 2 | 3 | 0 |
| | | | | | | | R.LPHRPVEDLAFAVAR.F | 99.70% | 47.2 | 39.7 | 37.9 | 0 | 0 | 3 | 0 |
| 1071a | 2-Phospho-D-glycerate hydrolase | gi\|533474 | 48340 | 100.00% | 12 | 34.50% | K.AIDENSCNALLLK.V | 99.70% | 74.4 | 43 | 48.2 | 0 | 3 | 0 | 0 |
| | | | | | | | R.IEEELGDKAVYAGANFR.R | 99.70% | 48 | 44.2 | 27.6 | 0 | 0 | 2 | 0 |
| | | | | | | | R.ISGEALKDLYK.S | 99.70% | 71.6 | 40.7 | 34 | 0 | 3 | 3 | 0 |
| | | | | | | | K.KAGWGVMASHR.S | 99.70% | 50.1 | 41.4 | 25 | 0 | 1 | 3 | 0 |

| | | | | | | | | | | | | | | | |
|---|---|---|---|---|---|---|---|---|---|---|---|---|---|---|---|
| | | | | | | | K.KAIDENSCNALLLK.V | 99.70% | 103 | 42.4 | 78 | 0 | 1 | 2 | 0 |
| | | | | | | | K.KIPLYQHIAEIAGNK.N | 99.70% | 42.9 | 37.3 | 24.5 | 0 | 0 | 1 | 0 |
| | | | | | | | K.MGSEVYHNLK.S | 99.70% | 59.4 | 41.7 | 40.8 | 0 | 3 | 0 | 0 |
| | | | | | | | K.SYDLNFKEENNDGSQR.I | 99.70% | 48 | 44.5 | 34.3 | 0 | 0 | 2 | 0 |
| | | | | | | | K.VNQIGSVTESIEAVK.M | 99.70% | 91.9 | 41.6 | 72.2 | 0 | 2 | 0 | 0 |
| | | | | | | | K.VQIVGDDLLVTNPK.R | 99.70% | 95 | 38.7 | 63.8 | 0 | 3 | 0 | 0 |
| | | | | | | | K.VQIVGDDLLVTNPKR.V | 98.70% | 30.5 | 37.4 | 9.52 | 0 | 0 | 1 | 0 |
| | | | | | | | K.YGQDATNVGDEGGFAPNIQENKEGLELLK.T | 99.70% | 87.7 | 45.5 | 0 | 0 | 0 | 2 | 0 |
| 1071b | Alpha-1,4-glucan-protein synthase | gi\|356495127 | 41712 | 100.00% | 9 | 27.40% | K.ASCISFK.D | 98.90% | 42 | 41.7 | 13.7 | 0 | 3 | 0 | 0 |
| | | | | | | | K.ASCISFKDSACR.C | 99.70% | 49.2 | 43 | 27.7 | 0 | 1 | 3 | 0 |
| | | | | | | | K.ASNPFVNLK.K | 98.90% | 41.5 | 41.7 | 18.2 | 0 | 1 | 0 | 0 |
| | | | | | | | R.CFGYMVSK.K | 98.60% | 39.1 | 40.9 | 18.3 | 0 | 3 | 0 | 0 |
| | | | | | | | R.EGAPTAVSHGLWLNIPDYDAPTQLVKPLER.N | 99.70% | 114 | 41.7 | 32.1 | 0 | 0 | 3 | 3 |
| | | | | | | | R.GYPFSLR.E | 94.90% | 31.2 | 41.5 | 11.7 | 0 | 1 | 0 | 0 |
| | | | | | | | K.VICDHLGLGVK.T | 99.70% | 76.6 | 41.8 | 57 | 0 | 3 | 3 | 0 |
| | | | | | | | R.YDDMWAGWCVK.V | 99.70% | 60.4 | 40.9 | 41.5 | 0 | 3 | 0 | 0 |
| | | | | | | | K.YIYTIDDDCFVAK.D | 99.70% | 56.7 | 44.2 | 43.2 | 0 | 2 | 0 | 0 |
| 1095 | Malate dehydrogenase | gi\|350536645 | 36152 | 100.00% | 4 | 15.00% | R.DDLFNINAGIVK.S | 99.70% | 57.4 | 42.3 | 36.9 | 0 | 1 | 0 | 0 |
| | | | | | | | K.RTQDGGTEVVEAK.A | 97.80% | 56.4 | 42.7 | 23.3 | 0 | 1 | 0 | 0 |
| | | | | | | | R.TQDGGTEVVEAK.A | 99.70% | 86.1 | 42.8 | 60.1 | 0 | 1 | 0 | 0 |
| | | | | | | | K.YCPNALVNMISNPVNSTVPIAAEVFKK.A | 99.70% | 62.3 | 43.4 | 46.7 | 0 | 0 | 1 | 0 |
| 1123a | Malate dehydrogenase | gi\|350536645 | 36152 | 100.00% | 5 | 21.70% | K.AGKGSMTLSIAYAGAIFADACLK.G | 99.70% | 74.9 | 45.8 | 60.2 | 0 | 0 | 1 | 0 |
| | | | | | | | R.DDLFNINAGIVK.S | 99.70% | 71.5 | 42.3 | 49.7 | 0 | 3 | 0 | 0 |
| | | | | | | | RTQDGGTEVVEAK.A | 99.70% | 53.5 | 43.1 | 19.7 | 0 | 1 | 0 | 0 |
| | | | | | | | R.TQDGGTEVVEAK.A | 99.70% | 85.4 | 42.8 | 56.7 | 0 | 2 | 0 | 0 |
| | | | | | | | K.YCPNALVNMISNPVNSTVPIAAEVFKK.A | 99.70% | 55.3 | 43.3 | 44.1 | 0 | 0 | 3 | 0 |
| 1123b | Proline iminopeptidase | gi\|224060965 | 37404 | 100.00% | 4 | 11.50% | K.GFFPSDSFLLENVDK.I | 99.70% | 65.6 | 44.3 | 46.8 | 0 | 3 | 0 | 0 |
| | | | | | | | R.IENHYFVNK.G | 99.70% | 49.1 | 42.3 | 21.8 | 0 | 2 | 0 | 0 |
| | | | | | | | R.IILFDQR.G | 98.60% | 35.5 | 39.3 | 0 | 0 | 1 | 0 | 0 |
| | | | | | | | K.VTGLVLR.G | 96.00% | 27.9 | 38.4 | 0 | 0 | 1 | 0 | 0 |
| 1153 | V-ATPase E subunit | gi\|3334410 | 26163 | 100.00% | 26 | 88.90% | K.ACVSHPEVIVDDIHLPPAPTSYDSHELSCSGGVVMASR.D | 98.50% | 33.5 | 46.5 | 18.2 | 0 | 0 | 0 | 2 |
| | | | | | | | K.AKQVDVRR.K | 96.40% | 30.2 | 39.6 | 8.56 | 0 | 1 | 0 | 0 |
| | | | | | | | K.ANEISVSAEEEFNIEKLQLVEAEK.K | 99.70% | 49.3 | 44.3 | 0 | 0 | 0 | 1 | 0 |
| | | | | | | | R.CREEDKHHVHR.V | 99.70% | 42.3 | 44.2 | 29 | 0 | 0 | 3 | 16 |
| | | | | | | | R.DGKIVFENTLDAR.L | 99.70% | 64.6 | 43.4 | 36.9 | 0 | 3 | 2 | 0 |
| | | | | | | | R.DGKIVFENTLDARLEVAFR.K | 99.50% | 38.5 | 41.9 | 24.2 | 0 | 0 | 1 | 0 |
| | | | | | | | K.EAASKELLLVSGDHHQYR.N | 99.70% | 54.3 | 43.9 | 44.3 | 0 | 0 | 1 | 0 |
| | | | | | | | K.ELIVQSLLR.L | 99.70% | 55.8 | 38.5 | 18.3 | 0 | 3 | 0 | 0 |
| | | | | | | | K.ELLLVSGDHHQYR.N | 99.70% | 77.4 | 43.7 | 43.9 | 0 | 3 | 2 | 0 |
| | | | | | | | K.ELLLVSGDHHQYRNLLKELIVQSLLR.L | 97.40% | 25.5 | 32.8 | 17.5 | 0 | 0 | 0 | 2 |
| | | | | | | | K.IEYSMQLNASR.I | 99.70% | 72.9 | 43 | 41.7 | 0 | 3 | 0 | 0 |
| | | | | | | | R.IKVLQAQDDLVNAMK.E | 99.70% | 100 | 40.6 | 0 | 0 | 5 | 3 | 0 |
| | | | | | | | R.IKVLQAQDDLVNAMKEAASK.E | 99.70% | 65.5 | 40.7 | 26.4 | 0 | 0 | 5 | 1 |
| | | | | | | | K.IRQEYERK.A | 99.30% | 35.8 | 41.3 | 14.3 | 0 | 0 | 1 | 0 |
| | | | | | | | K.IVFENTLDAR.L | 99.70% | 68 | 42.1 | 34.3 | 0 | 3 | 0 | 0 |
| | | | | | | | R.KIEYSMQLNASR.I | 99.70% | 97.8 | 43.1 | 81.6 | 0 | 3 | 3 | 0 |
| | | | | | | | R.KKLPQIR.K | 99.00% | 28.7 | 30.1 | 0 | 0 | 2 | 0 | 0 |
| | | | | | | | R.LEVAFR.K | 98.40% | 35.4 | 39.7 | 0 | 0 | 2 | 0 | 0 |
| | | | | | | | R.LEVAFRK.K | 99.00% | 37.6 | 39.4 | 14.1 | 0 | 3 | 0 | 0 |
| | | | | | | | R.LKEPAVLLR.C | 99.70% | 50.2 | 30.5 | 0 | 0 | 2 | 0 | 0 |
| | | | | | | | K.LQLVEAEKK.K | 98.40% | 35.8 | 40.1 | 0 | 0 | 1 | 0 | 0 |
| | | | | | | | R.NLLKELIVQSLLR.L | 99.70% | 69.8 | 31.8 | 33.8 | 0 | 3 | 3 | 0 |
| | | | | | | | R.QEAEEKANEISVSAEEEFNIEKLQLVEAEK.K | 99.70% | 49.7 | 45.3 | 0 | 0 | 0 | 0 | 2 |
| | | | | | | | R.RKIEYSMQLNASR.I | 99.70% | 60.4 | 42.6 | 41.3 | 0 | 1 | 5 | 2 |

| | | | | | | | | | | | | | | | |
|---|---|---|---|---|---|---|---|---|---|---|---|---|---|---|---|
| | | | | | | | R.VLHSAREEYGEK.A | 99.70% | 57.8 | 43.1 | 42.5 | 0 | 3 | 0 | 0 |
| | | | | | | | K.VLQAQDDLVNAMK.E | 99.70% | 90.6 | 42.9 | 0 | 0 | 6 | 0 | 0 |
| 1307 | Ascorbate peroxidase | gi\|239586448 | 18421 | 100.00% | 5 | 41.10% | K.ALLSDPAFRPLVEK.Y | 99.70% | 63 | 39.2 | 38.6 | 0 | 2 | 0 | 0 |
| | | | | | | | K.ELLSGEKEGLLQLPSDK.A | 99.70% | 51.4 | 41.3 | 0 | 0 | 0 | 2 | 0 |
| | | | | | | | K.ELLSGEKEGLLQLPSDKALLSDPAFRPLVEK.Y | 99.70% | 42.2 | 36.4 | 0.88 | 0 | 0 | 0 | 1 |
| | | | | | | | K.QMGLSDKDIVALSGAHTLGR.C | 99.70% | 59.8 | 43.6 | 28.6 | 0 | 0 | 3 | 2 |
| | | | | | | | K.YAADEDAFFADYAEAHLK.L | 99.70% | 64.6 | 47.2 | 51.1 | 0 | 1 | 3 | 0 |
| 1325 | Fructose-biphosphate aldolase | gi\|2213867 | 38158 | 100.00% | 8 | 20.70% | K.ALNDHHVLLEGTLLKPNMVTPGSESK.K | 99.70% | 62.1 | 42.3 | 49 | 0 | 0 | 0 | 3 |
| | | | | | | | K.ALNDHHVLLEGTLLKPNMVTPGSESKK.V | 99.30% | 37 | 40.9 | 29.3 | 0 | 0 | 0 | 0 |
| | | | | | | | R.CAEVTER.V | 96.80% | 30.6 | 39.2 | 5.56 | 0 | 3 | 0 | 0 |
| | | | | | | | R.CAQYYAAGAR.F | 99.70% | 67.6 | 42 | 44 | 0 | 3 | 0 | 0 |
| | | | | | | | K.KPWTLSFSYGR.A | 98.80% | 42.9 | 44.2 | 21.2 | 0 | 1 | 3 | 0 |
| | | | | | | | K.KVAPEVIAEYTVR.A | 99.70% | 54.6 | 40.8 | 35 | 0 | 2 | 3 | 0 |
| | | | | | | | K.VAPEVIAEYTVR.A | 99.70% | 61.7 | 41.7 | 36.9 | 0 | 2 | 0 | 0 |
| | | | | | | | R.VLAACYK.A | 98.70% | 40 | 42.1 | 0 | 0 | 2 | 0 | 0 |
| 1501 | Alpha-galactosidase-like protein | gi\|297816956 | 47551 | 100.00% | 3 | 10.70% | K.APLLIGCDVR.N | 99.70% | 52.7 | 39.6 | 0 | 0 | 2 | 0 | 0 |
| | | | | | | | K.EVIAVNQDPLGVQGR.K | 99.70% | 82.9 | 41.4 | 67.3 | 0 | 1 | 1 | 0 |
| | | | | | | | R.TTDDINDTWASMTTIADLNNK.W | 99.50% | 53.6 | 45.8 | 0 | 0 | 0 | 1 | 0 |
| 1502 | 5-Methyltetrahydropteroyltri-glutamate-homocysteine methyltransferase | gi\|8134568 | 84824 | 100.00% | 62 | 66.10% | K.AATALKGSDHR.R | 97.40% | 39 | 40.3 | 17.3 | 0 | 1 | 0 | 0 |
| | | | | | | | K.AGINVIQIDEAALR.E | 99.70% | 73.4 | 39 | 45.4 | 0 | 3 | 3 | 0 |
| | | | | | | | K.AGINVIQIDEAALREGLPLR.K | 99.70% | 47.4 | 37 | 33.7 | 0 | 0 | 3 | 0 |
| | | | | | | | K.AGINVIQIDEAALREGLPLRK.S | 99.70% | 56.2 | 33.5 | 52 | 0 | 0 | 2 | 3 |
| | | | | | | | K.AIKEEISKVVK.L | 99.70% | 44.4 | 34.2 | 31.6 | 0 | 3 | 3 | 0 |
| | | | | | | | K.AKKISEEEYVK.A | 99.70% | 52.4 | 41.1 | 39.7 | 0 | 0 | 2 | 0 |
| | | | | | | | K.ALGVDTVPVLVGPVSYLLLSK.Q | 99.70% | 63.2 | 31.2 | 48.7 | 0 | 3 | 6 | 0 |
| | | | | | | | K.ALSGQKDEAFFSANAAALASR.K | 99.70% | 112 | 44.4 | 97.7 | 0 | 3 | 2 | 1 |
| | | | | | | | K.ALSGQKDEAFFSANAAALASRK.S | 99.70% | 82.5 | 43.1 | 68.7 | 0 | 0 | 3 | 1 |
| | | | | | | | R.ATTVSSRLDAQQK.K | 98.60% | 48.3 | 42.3 | 19.2 | 0 | 2 | 0 | 0 |
| | | | | | | | K.AVLEYK.E | 99.20% | 43.5 | 36.4 | 0 | 0 | 3 | 0 | 0 |
| | | | | | | | K.AVLEYKEAK.A | 99.70% | 64.4 | 40.5 | 0 | 0 | 3 | 0 | 0 |
| | | | | | | | K.DEAFFSANAAALASR.K | 99.70% | 89.5 | 44.9 | 0 | 0 | 1 | 0 | 0 |
| | | | | | | | R.EGLPLRK.S | 96.70% | 33.5 | 36.4 | 10.9 | 0 | 2 | 0 | 0 |
| | | | | | | | R.EGVKYGAGIGPGVYDIHSPR.I | 99.70% | 65.5 | 44.2 | 58 | 0 | 0 | 3 | 0 |
| | | | | | | | K.FALESFWDGK.S | 99.70% | 57 | 42.4 | 37.2 | 0 | 3 | 0 | 0 |
| | | | | | | | K.FALESFWDGKSTAEDLK.K | 99.70% | 43.2 | 44.9 | 2.02 | 0 | 0 | 1 | 0 |
| | | | | | | | K.FALESFWDGKSTAEDLKK.V | 99.70% | 87.2 | 44.4 | 72.4 | 0 | 1 | 3 | 1 |
| | | | | | | | K.GMLTGPVTILNWSFVR.N | 99.70% | 111 | 41.8 | 0 | 0 | 6 | 1 | 0 |
| | | | | | | | R.GNASVPAMEMTK.W | 99.70% | 72.4 | 42 | 24.2 | 0 | 10 | 0 | 0 |
| | | | | | | | R.GTKTLDLVKAEFPSGK.Y | 99.70% | 39.7 | 40 | 20.6 | 0 | 0 | 1 | 0 |
| | | | | | | | K.GVDKSFDLLSLLPK.I | 99.70% | 55.8 | 38.3 | 12.6 | 0 | 2 | 3 | 0 |
| | | | | | | | K.GVTGYGFDLVR.G | 99.70% | 79.1 | 41.6 | 57.2 | 0 | 3 | 0 | 0 |
| | | | | | | | R.HETCYQIALAIKNEVEDLEK.A | 99.70% | 71 | 44.6 | 58.4 | 0 | 1 | 3 | 3 |
| | | | | | | | R.HETCYQIALAIKNEVEDLEKAGINVIQIDEAALR.E | 99.70% | 71.3 | 41.8 | 62.8 | 0 | 0 | 0 | 2 |
| | | | | | | | K.ILPIYKEVVAELK.E | 99.70% | 50.2 | 32.7 | 34.5 | 0 | 3 | 3 | 0 |
| | | | | | | | R.IPPTEELADR.I | 99.70% | 77 | 41.2 | 0 | 0 | 4 | 0 | 0 |
| | | | | | | | R.IPPTEELADRIR.K | 99.30% | 48 | 40.1 | 0 | 0 | 3 | 0 | 0 |
| | | | | | | | K.ISEEEYVK.A | 99.70% | 59.1 | 43 | 36.9 | 0 | 3 | 0 | 0 |
| | | | | | | | K.KISEEEYVK.A | 99.70% | 51.6 | 42.2 | 17.9 | 0 | 3 | 3 | 0 |
| | | | | | | | K.KLNLPILPTTTIGSFPQTVELR.R | 99.70% | 68.9 | 33.4 | 11.8 | 0 | 3 | 1 | 0 |
| | | | | | | | K.KLNLPILPTTTIGSFPQTVELRR.V | 99.70% | 35.6 | 31.1 | 0 | 0 | 0 | 3 | 0 |
| | | | | | | | R.KMLAVLESNVLWVNPDCGLK.T | 99.70% | 81.6 | 42.9 | 69.6 | 0 | 3 | 3 | 0 |

| | | | | | | | | | | | | | | | |
|---|---|---|---|---|---|---|---|---|---|---|---|---|---|---|---|
| | | | | | | | R.KSEHDFYLK.W | 99.70% | 58.7 | 43.3 | 28.5 | 0 | 3 | 0 | 0 |
| | | | | | | | R.KYGEVNPALSNMVAAAK.Q | 99.70% | 106 | 42.7 | 82 | 0 | 6 | 6 | 0 |
| | | | | | | | K.LDDEIKSWLAFAAQK.V | 99.70% | 44.1 | 43.1 | 0 | 0 | 0 | 1 | 0 |
| | | | | | | | K.LLSVFR.E | 99.00% | 40.7 | 35.4 | 0 | 0 | 3 | 0 | 0 |
| | | | | | | | K.LLSVFREGVK.Y | 99.70% | 60.2 | 37 | 0 | 0 | 1 | 0 | 0 |
| | | | | | | | K.LNLPILPTTTIGSFPQTVELR.R | 99.70% | 66.5 | 35.5 | 10.4 | 0 | 0 | 2 | 0 |
| | | | | | | | K.LNLPILPTTTIGSFPQTVELRR.V | 99.70% | 41.6 | 33.9 | 0.99 | 0 | 0 | 2 | 0 |
| | | | | | | | K.LQEELDIDVLVHGEPER.N | 99.70% | 69.3 | 44.1 | 0 | 0 | 0 | 3 | 0 |
| | | | | | | | K.MLAVLESNVLWVNPDCGLK.T | 99.70% | 65.4 | 44 | 45.9 | 0 | 3 | 3 | 0 |
| | | | | | | | R.NDQPRHETCYQIALAIK.N | 99.70% | 62.7 | 44.8 | 46.9 | 0 | 0 | 3 | 0 |
| | | | | | | | R.NDQPRHETCYQIALAIKNEVEDLEK.A | 99.70% | 74.6 | 45.8 | 49.3 | 0 | 0 | 2 | 3 |
| | | | | | | | R.NIWANDLAASLATLEALEGVVGK.D | 99.70% | 52.4 | 40.8 | 43.6 | 0 | 0 | 1 | 0 |
| | | | | | | | R.NIWANDLAASLATLEALEGVVGKDK.L | 99.70% | 130 | 40.4 | 90.6 | 0 | 2 | 3 | 0 |
| | | | | | | | K.QLRQELASAK | 99.70% | 61 | 40.9 | 37 | 0 | 3 | 3 | 0 |
| | | | | | | | K.QMADAGIK.Y | 99.60% | 59.2 | 41 | 18.6 | 0 | 6 | 0 | 0 |
| | | | | | | | R.RATTVSSR.L | 96.00% | 31.7 | 40.6 | 7.64 | 0 | 6 | 0 | 0 |
| | | | | | | | R.SDEKLLSVFR.E | 99.70% | 64 | 41.6 | 0 | 0 | 3 | 3 | 0 |
| | | | | | | | K.SEHDFYLK.W | 97.90% | 41.7 | 41.5 | 24.5 | 0 | 3 | 0 | 0 |
| | | | | | | | K.SFDLLSLLPK.I | 99.70% | 68.2 | 39.3 | 40 | 0 | 3 | 0 | 0 |
| | | | | | | | K.SWLAFAAQK.V | 99.70% | 55.9 | 46 | 0 | 0 | 2 | 0 | 0 |
| | | | | | | | K.TLDLVKAEFPSGK.Y | 99.70% | 83.5 | 43.7 | 65.9 | 0 | 3 | 3 | 0 |
| | | | | | | | K.TLDLVKAEFPSGKYLFAGVVDGR.N | 99.70% | 53.1 | 40.1 | 29.9 | 0 | 0 | 0 | 1 |
| | | | | | | | K.TLISLKGVTGYGFDLVR.G | 99.70% | 76.3 | 37.9 | 63.4 | 0 | 1 | 3 | 0 |
| | | | | | | | K.VLEVNALAK.A | 99.70% | 54.8 | 40.2 | 5.3 | 0 | 2 | 0 | 0 |
| | | | | | | | K.WFDTNYHFIVPELGPEVNFSYASHK.A | 99.70% | 75.4 | 45.9 | 19.2 | 0 | 0 | 3 | 0 |
| | | | | | | | K.YGAGIGPGVYDIHSPR.I | 99.70% | 105 | 44 | 85.7 | 0 | 3 | 3 | 0 |
| | | | | | | | K.YGEVNPALSNMVAAAK.Q | 99.70% | 90.5 | 43.6 | 76.2 | 0 | 6 | 0 | 0 |
| | | | | | | | K.YIPSNTFSYYDQVLDTTAMLGAVPPR.Y | 99.00% | 47.8 | 45.4 | 3.41 | 0 | 0 | 1 | 0 |
| | | | | | | | K.YLFAGVVDGR.N | 99.70% | 55.1 | 42.2 | 24.6 | 0 | 3 | 0 | 0 |
| 1503 | Triosephosphate isomerase | gi\|388507284 | 33452 | 100.00% | 5 | 23.10% | K.FFVGGNWK.C | 95.50% | 31.9 | 41.3 | 12.9 | 0 | 3 | 0 | 0 |
| | | | | | | | R.HVIGEKDEFIGK.K | 99.70% | 47.1 | 41.7 | 35.3 | 0 | 1 | 2 | 0 |
| | | | | | | | R.HVIGEKDEFIGKK.A | 99.70% | 47 | 40.7 | 32.9 | 0 | 1 | 3 | 3 |
| | | | | | | | K.KAAYALSEGLGVIACIGELLEER.E | 99.70% | 67.9 | 41.2 | 54.3 | 0 | 0 | 1 | 0 |
| | | | | | | | K.KEDIDGFLVGGASLKGPEFATIVNSVTSK.K | 99.70% | 46.4 | 41 | 1.71 | 0 | 0 | 0 | 3 |
| 1508 | V-ATPase A subunit | gi\|13235340 | 68900 | 100.00% | 54 | 85.90% | K.AREVLQREDDLNEIVQLVGK.D | 99.70% | 70.2 | 39.5 | 53.1 | 0 | 0 | 3 | 2 |
| | | | | | | | R.DMGYNVSMMADSTSR.W | 99.70% | 107 | 41.6 | 14.4 | 0 | 14 | 4 | 0 |
| | | | | | | | R.EASIYTGITIAEYFR.D | 99.70% | 106 | 42.8 | 0 | 0 | 3 | 3 | 0 |
| | | | | | | | R.EASIYTGITIAEYFRDMGYNVSMMADSTSR.W | 99.70% | 70.6 | 46.9 | 0 | 0 | 0 | 4 | 2 |
| | | | | | | | R.EDDLNEIVQLVGK.D | 99.70% | 88.4 | 43.6 | 69.4 | 0 | 2 | 0 | 0 |
| | | | | | | | K.EFTMLQSWPVR.T | 99.20% | 53.7 | 42.7 | 33.3 | 0 | 5 | 0 | 0 |
| | | | | | | | R.EVLQREDDLNEIVQLVGK.D | 99.70% | 56.2 | 45.4 | 35.8 | 0 | 3 | 3 | 0 |
| | | | | | | | K.FDPDFINIR.T | 99.40% | 54.7 | 41.9 | 32.1 | 0 | 3 | 0 | 0 |
| | | | | | | | K.FEDPAEGEDTLVAK.F | 99.70% | 96.2 | 43.2 | 44.3 | 0 | 3 | 0 | 0 |
| | | | | | | | K.FQKLYEDLSAAFR.N | 99.70% | 47.8 | 42.5 | 23.4 | 0 | 3 | 3 | 0 |
| | | | | | | | R.GAGSDGQKITYSLIK.L | 99.70% | 75 | 40.6 | 20.7 | 0 | 3 | 3 | 0 |
| | | | | | | | R.GAGSDGQKITYSLIKLR.L | 99.70% | 45.4 | 37.4 | 31.5 | 0 | 0 | 1 | 0 |
| | | | | | | | R.GNEMAEVLMDFPQLTMTLPDGR.E | 99.70% | 90.5 | 46.2 | 77.7 | 0 | 2 | 0 | 0 |
| | | | | | | | R.GNEMAEVLMDFPQLTMTLPDGREESVMK.R | 99.70% | 74.2 | 46.4 | 57.7 | 0 | 0 | 3 | 0 |
| | | | | | | | R.GNEMAEVLMDFPQLTMTLPDGREESVMKR.T | 99.70% | 63.8 | 46.3 | 37 | 0 | 0 | 4 | 2 |
| | | | | | | | R.GVAVPPLDKDALWEFEPK.K | 99.70% | 44.4 | 41.4 | 32.4 | 0 | 3 | 3 | 0 |
| | | | | | | | R.GVAVPPLDKDALWEFEPKK.I | 98.50% | 30.2 | 39.3 | 17 | 0 | 0 | 2 | 0 |
| | | | | | | | K.HFPSVNWLISYSK.Y | 99.70% | 48.8 | 42.4 | 21.5 | 0 | 3 | 3 | 0 |
| | | | | | | | K.ITYIAPPGQYSIQDTVLELEFQGVK.K | 99.70% | 49.3 | 40.1 | 0 | 0 | 0 | 2 | 0 |
| | | | | | | | K.ITYIAPPGQYSIQDTVLELEFQGVKK.E | 99.70% | 48.5 | 38.5 | 0 | 0 | 0 | 2 | 1 |
| | | | | | | | K.KEFTMLQSWPVR.T | 99.70% | 71.6 | 42.7 | 55.1 | 0 | 2 | 2 | 0 |

| | | | | | | | | |
|---|---|---|---|---|---|---|---|---|
| R.KHFPSVNWLISYSK.Y | 99.70% | 76.7 | 41 | 53.4 | 0 | 3 | 3 | 1 |
| K.KIGEGDLLTGGDLYAK.V | 99.70% | 75.3 | 41.2 | 57.4 | 0 | 3 | 3 | 0 |
| R.KVSGPVVVADGMNGAAMYELVR.V | 99.70% | 66.9 | 43.8 | 32.2 | 0 | 1 | 7 | 0 |
| K.LAADTPLLTGQR.V | 99.70% | 83.1 | 38.9 | 60.9 | 0 | 3 | 3 | 0 |
| R.LAEMPADSGYPAYLAAR.L | 99.70% | 104 | 44 | 80.6 | 0 | 3 | 2 | 0 |
| R.LASFYER.A | 99.10% | 49.7 | 40.5 | 16.9 | 0 | 2 | 0 | 0 |
| R.LEGDSATIQVYEETAGLMVNDPVLR.T | 99.70% | 75.6 | 44.5 | 63.4 | 0 | 1 | 3 | 0 |
| R.LGDLFYR.L | 99.40% | 53.5 | 40.9 | 15.5 | 0 | 3 | 0 | 0 |
| K.LLREDYLAQNAFTPYDK.F | 99.70% | 44.5 | 43.9 | 29.1 | 0 | 0 | 1 | 0 |
| K.LRLGDLFYR.L | 99.70% | 34.4 | 37.8 | 9.32 | 0 | 1 | 3 | 0 |
| R.LTTFEDSEKESEYGYIR.K | 99.70% | 76.8 | 45 | 67.5 | 0 | 3 | 1 | 0 |
| R.LTTFEDSEKESEYGYIRK.V | 99.40% | 49 | 45.5 | 38.7 | 0 | 0 | 3 | 0 |
| R.LVSQKFEDPAEGEDTLVAK.F | 99.70% | 53.5 | 43.2 | 21.9 | 0 | 3 | 3 | 0 |
| K.LYEDLSAAFR.N | 99.70% | 76.4 | 45.5 | 34 | 0 | 3 | 0 | 0 |
| M.PAVYGDRLTTFEDSEKESEYGYIR.K | 99.40% | 42.5 | 45.6 | 30.1 | 0 | 0 | 0 | 2 |
| K.RSGDVYIPR.G | 98.90% | 45.2 | 41.5 | 20.7 | 0 | 3 | 0 | 0 |
| K.RTTLVANTSNMPVAAR.E | 99.70% | 83.7 | 42.4 | 63 | 0 | 1 | 4 | 0 |
| R.SGDVYIPR.G | 99.10% | 55.1 | 40.5 | 21.5 | 0 | 3 | 0 | 0 |
| R.TGSVTIVGAVSPPGGDFSDPVTSATLSIVQVFWGLDKK.L | 99.70% | 68.9 | 38.4 | 58.2 | 0 | 0 | 1 | 0 |
| R.THKPLSVELGPGILGNIFDGIQRPLK.T | 99.70% | 63.6 | 30.9 | 62 | 0 | 0 | 2 | 4 |
| R.THKPLSVELGPGILGNIFDGIQRPLKTIAK.R | 99.70% | 56.6 | 31.9 | 45.8 | 0 | 0 | 0 | 2 |
| R.THKPLSVELGPGILGNIFDGIQRPLKTIAKR.S | 99.70% | 56.1 | 34.3 | 52.6 | 0 | 0 | 0 | 3 |
| R.TTLVANTSNMPVAAR.E | 99.70% | 101 | 42.9 | 76 | 0 | 6 | 6 | 0 |
| K.TVISQALSK.Y | 99.70% | 61 | 39.4 | 31.3 | 0 | 3 | 0 | 0 |
| K.TVISQALSKYSNSDAVVYVGCGER.G | 99.70% | 56.2 | 44.2 | 8.33 | 0 | 0 | 2 | 0 |
| R.VGYDNLIGEIIR.L | 99.70% | 84.9 | 40.3 | 57.7 | 0 | 3 | 2 | 0 |
| R.VGYDNLIGEIIRLEGDSATIQVYEETAGLMVNDPVLR.T | 99.70% | 82.3 | 40.9 | 70.1 | 0 | 0 | 0 | 1 |
| K.VKCLGGPER.T | 96.60% | 39.1 | 42.2 | 5.59 | 0 | 1 | 0 | 0 |
| R.VLDALFPSVLGGTCAIPGAFGCGK.T | 99.70% | 104 | 43.3 | 90.1 | 0 | 3 | 3 | 0 |
| K.VSGPVVVADGMNGAAMYELVR.V | 99.70% | 117 | 43.7 | 98.7 | 0 | 3 | 5 | 0 |
| R.WAEALREISGR.L | 98.00% | 46.6 | 41.6 | 23.1 | 0 | 3 | 0 | 0 |
| K.YSNSDAVVYVGCGER.G | 99.70% | 84.1 | 43.5 | 11.8 | 0 | 3 | 0 | 0 |
| K.YTSALETFYEKFDPDFINIR.T | 99.70% | 67.1 | 44.5 | 59.7 | 0 | 1 | 3 | 0 |
