# Supplementary material for: Single-cell-type quantitative proteomic and ionomic analysis of epidermal bladder cells from the halophyte model plant Mesembryanthemum crystallinum to identify salt-responsive proteins
Source: BMC Plant Biol. 2016 May 10;16:110. doi: 10.1186/s12870-016-0797-1 (PMC4862212; doi:10.1186/s12870-016-0797-1)

Additional file 3. SDS-PAGE separated total proteins isolated from EBC extract. Total EBC protein from three biological replicates for control (left gel) and salt-treated (right gel) were separated on 10% SDS-PAGE mini-gels and stained with Coomassie Brilliant Blue R-250 (CBB) dye. Letters on the right represent the gel pieces that were subjected to protein identification by LC-MS/MS. Numbers on the left are protein molecular masses (kDa) determined by molecular weight markers. The protein band corresponding to cysteine protease is indicated with the * symbol.

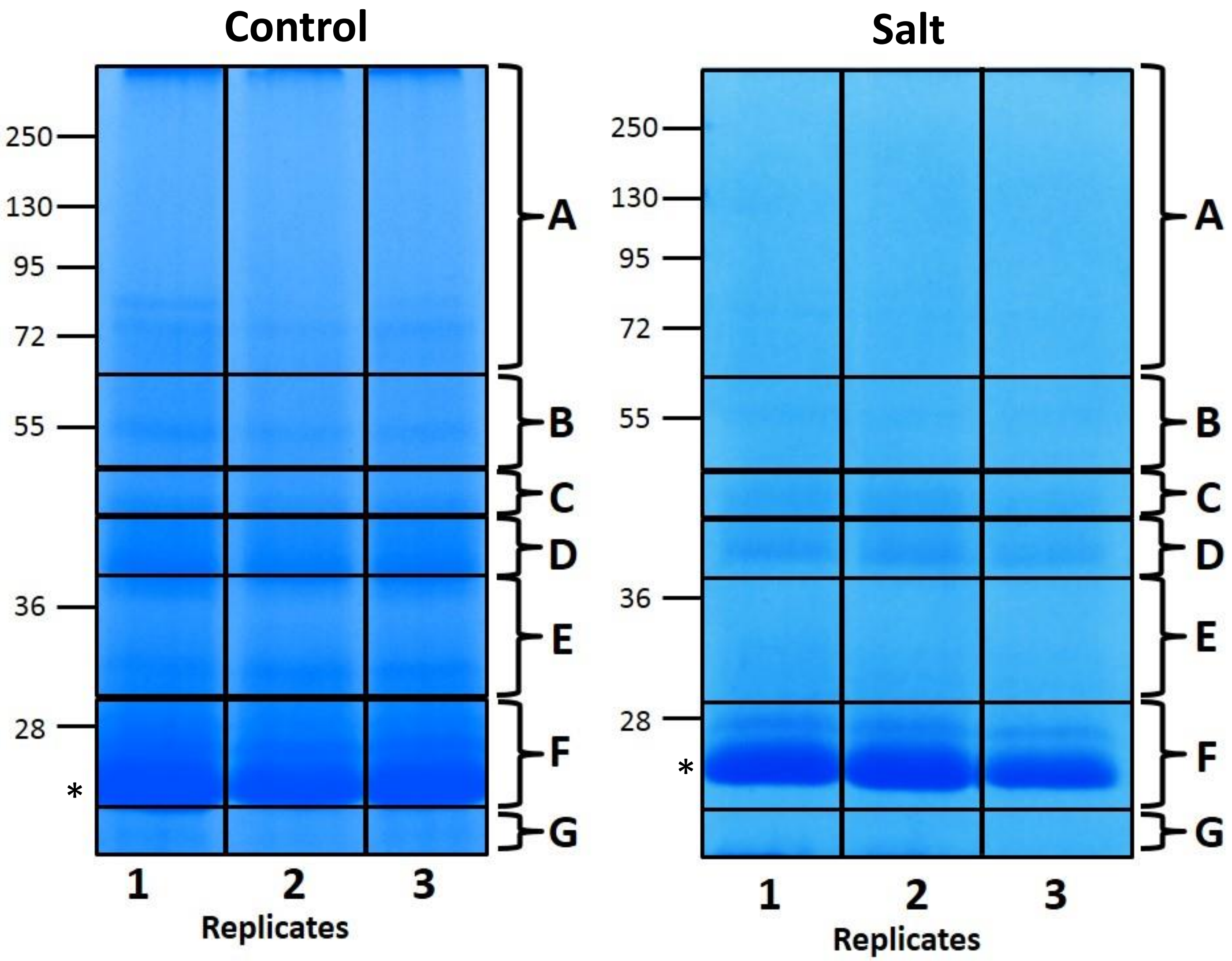

Supplement: Additional file 3: — Figure showing the 1D-SDS-PAGE separated total proteins isolated from EBC used for GeLC-MS/MS. (PDF 263 kb) [file 12870_2016_797_MOESM3_ESM.pdf]
