# Supplementary material for: Single-cell-type quantitative proteomic and ionomic analysis of epidermal bladder cells from the halophyte model plant Mesembryanthemum crystallinum to identify salt-responsive proteins
Source: BMC Plant Biol. 2016 May 10;16:110. doi: 10.1186/s12870-016-0797-1 (PMC4862212; doi:10.1186/s12870-016-0797-1)
Supplement: Additional file 4: — Table of the ionomics data to quantify elements in the EBC extract from control and salt-treated plants. (PDF 343 kb) [file 12870_2016_797_MOESM4_ESM.pdf]

Additional file 4: Elements measured in EBC extracts

ELEMENT	Sample (mg/L)											
	Control						Salt					
	1	2	3	4	5	6	1	2	3	4	5	6
Silver	0.000	0.000	0.000	0.000	0.001	0.000	0.000	0.001	0.000	0.000	0.001	0.000
Aluminium	0.131	0.038	0.074	0.040	0.108	0.059	0.042	0.050	0.048	0.035	0.052	0.050
Arsenic	0.000	0.000	0.000	0.000	0.000	0.000	0.001	0.000	0.000	0.000	0.000	0.001
Cadmium	0.002	0.001	0.001	0.000	0.001	0.001	0.001	0.001	0.001	0.001	0.001	0.001
Chromium	0.008	0.002	0.004	0.005	0.007	0.003	0.005	0.003	0.001	0.002	0.004	0.004
Copper	0.411	0.215	0.290	0.210	0.318	0.294	0.330	0.240	0.212	0.204	0.227	0.239
Iron	0.178	0.076	0.165	0.073	0.283	0.133	0.313	0.128	0.080	0.103	0.118	0.133
Manganese	0.278	0.595	0.064	0.195	0.072	0.248	1.772	1.206	0.837	0.939	0.747	1.005
Nickel	0.055	0.007	0.021	0.011	0.012	0.011	0.015	0.010	0.005	0.009	0.009	0.009
Lead	0.006	0.002	0.002	0.001	0.002	0.001	0.002	0.002	0.001	0.001	0.002	0.001
Selenium	0.004	0.012	0.016	0.002	0.014	0.011	0.026	0.024	0.011	0.007	0.007	0.006
Zinc	0.231	0.191	0.109	0.155	0.159	0.159	3.143	0.445	0.257	0.326	0.425	0.279
Mercury	<0.005	<0.005	<0.005	<0.005	<0.005	<0.005	<0.005	<0.005	<0.005	<0.005	<0.005	<0.005
Boron	0.720	0.532	0.413	0.614	0.368	0.584	0.785	0.681	0.386	0.434	0.364	0.349
Silicon	10.06	20.89	8.89	64.76	8.21	8.95	6.76	6.35	3.97	12.42	14.36	5.91
Vanadium	0.002	0.001	0.001	0.001	0.001	0.001	0.005	0.004	0.004	0.002	0.003	0.004
Cobalt	0.001	0.001	0.001	0.001	0.001	0.001	0.001	0.001	0.000	0.000	0.000	0.001
Molybdenum	0.310	0.386	0.714	0.406	0.923	0.725	1.134	0.336	0.123	0.074	0.069	0.058
Barium	0.160	0.020	0.035	0.013	0.030	0.021	0.051	0.020	0.021	0.021	0.051	0.014
Calcium	1.74	2.55	0.87	1.86	1.22	1.24	1.31	1.14	0.97	1.40	0.83	1.02
Magnesium	194	426	155	285	196	191	164	181	146	149	105	141
Potassium	8,947	5,897	8,466	5,701	8,874	6,687	2,617	2,640	1,291	895	1,341	1,040
Sodium	531	1,014	433	396	497	471	13,766	11,586	11,545	11,094	9,521	12,563

Chloride	3,397	3,533	3,450	2,505	3,399	2,582			21,384	18,607	17,774	16,929	14,557	18,799		
Sulfur	495	440	453	355	432	474			142	138	102	103	85	95		
Phosphorus	5.76	5.53	5.51	5.49	6.99	8.36			9.08	7.03	8.32	7.10	8.30	10.16		
Bromide	10.75	9.73	12.39	9.14	12.60	10.38			19.56	15.09	9.69	7.23	6.20	7.17		
