# Supplementary material for: Single-cell-type quantitative proteomic and ionomic analysis of epidermal bladder cells from the halophyte model plant Mesembryanthemum crystallinum to identify salt-responsive proteins
Source: BMC Plant Biol. 2016 May 10;16:110. doi: 10.1186/s12870-016-0797-1 (PMC4862212; doi:10.1186/s12870-016-0797-1)
Supplement: Additional file 5: — Table listing the experimental design for the 2D-DIGE analysis. (PDF 96 kb) [file 12870_2016_797_MOESM5_ESM.pdf]

Additional file 5. DIGE experimental design.

	CyDye			
**Gel #**	**Cy3**	**Cy5**	**Cy2 (internal standard pool)**	**Protein/IEF gel strip**
1	50 µg BC1	50 µg BS1	6.25 µg each of BC1+BC2+BC3+BC4 BS1+BS2+BS3+BS4	150 µg
2	50 µg BC2	50 µg BS2	6.25 µg each of BC1+BC2+BC3+BC4 BS1+BS2+BS3+BS4	150 µg
3	50 µg BS3	50 µg BC3	6.25 µg each of BC1+BC2+BC3+BC4 BS1+BS2+BS3+BS4	150 µg
4	50 µg BS4	50 µg BC4	6.25 µg each of BC1+BC2+BC3+BC4 BS1+BS2+BS3+BS4	150 µg

BC1 – control sample 1, BC2 – control sample 2, BC3 – control sample 3, BC4 – control sample 4
BS1 – salt-treated sample 1, BS2 - salt-treated sample 2, BS3 - salt-treated sample 3, BS4 – salt-treated sample 4
