# Supplementary material for: Single-cell-type quantitative proteomic and ionomic analysis of epidermal bladder cells from the halophyte model plant Mesembryanthemum crystallinum to identify salt-responsive proteins
Source: BMC Plant Biol. 2016 May 10;16:110. doi: 10.1186/s12870-016-0797-1 (PMC4862212; doi:10.1186/s12870-016-0797-1)
Supplement: Additional file 6: — Table listing the gel scanning parameters used for the Typhoon Imager. (PDF 7 kb) [file 12870_2016_797_MOESM6_ESM.pdf]

Additional file 6. Gel Scanning Parameters used for the Typhoon scanner.

CyDye	Emission filter (nm) /Band Pass	Laser (nm)	Resolution (μm)	PMT[a] (V)
Cy 2	520/40	Blue 488	100	600
Cy 3	580/30	Green 532	100	600
Cy 5	670/30	Red 633	100	600

[a]Photomultiplier tube voltage
